# Reaction of Aldoximes with Sodium Chloride and Oxone under Ball-Milling Conditions

**DOI:** 10.3390/molecules25163719

**Published:** 2020-08-14

**Authors:** Kuan Chen, Chuang Niu, Guan-Wu Wang

**Affiliations:** 1 Hefei National Laboratory for Physical Sciences at the Microscale and Department of Chemistry, University of Science and Technology of China, Hefei 230026, Anhui, China; kuanc@mail.ustc.edu.cn (K.C.); cniu@mail.ustc.edu.cn (C.N.); 2 State Key Laboratory of Applied Organic Chemistry, Lanzhou University, Lanzhou 730000, Gansu, China

**Keywords:** mechanochemistry, aldoximes, sodium chloride, Oxone, *N*-acyloxyimidoyl chlorides

## Abstract

The solvent-free mechanochemical reaction has aroused increasing interest among scientists. Mechanical ball-milling can implement reactions under mild conditions, shorten the reaction time, and improve the reaction efficiency. Particularly, the most attractive characteristic of mechanochemistry is that it can alter the reaction pathway. However, few such examples have been reported so far. In this paper, we report the reaction of aldoximes with NaCl and Oxone under ball-milling conditions to afford *N*-acyloxyimidoyl chlorides, which are different from those of the liquid-phase counterpart.

## 1. Introduction

Since the rise of modern chemistry in the 17th century, traditional solution-based methods have dominated in most synthetic research laboratories and industries. Nevertheless, liquid-phase reactions are characterized by using bulk toxic organic solvents and harsh reaction conditions. With the need for cleaner, safer, and more sustainable chemical transformations, the use of mechanochemistry, which is identified by IUPAC as one of the 10 world-changing technologies [1] in chemical synthesis, has boomed over the past few decades and is rapidly becoming a powerful tool for environmentally friendly and sustainable synthesis of molecules and materials, including coordination, supramolecular, and covalent structures [2,3,4,5,6,7,8,9,10,11,12,13,14].

**Scheme 1:**
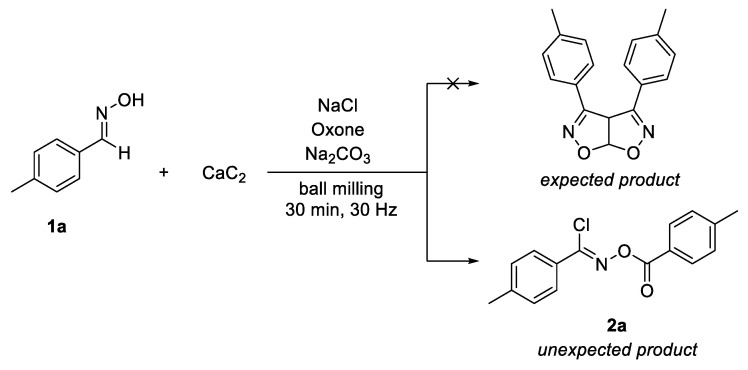
The unexpected product **2a** obtained from **1a** without participation of CaC_2_.

The mechanical ball-milling technique, an eco-friendly mechanochemical protocol, can not only promote solvent-free reactions and increase the reaction efficiency [6,7,8,9,10,11,12], even more attractively, but also alter chemical reactivity and selectivity compared to the solution-based counterparts, leading to different products, which draws forth the tantalizing hypothesis that some molecules can only be obtained by mechanochemistry [13,14]. For instance, Wang et al. reported the reaction of [60]fullerene (C_60_) and KCN under high-speed vibration ball milling (HSVM) for 30 min to unexpectedly afford fullerene dimer C_120_ even after quenching with trifluoroacetic acid (TFA) [15], while the same reaction in a mixture of 1,2-dichlorobenzene (ODCB) and *N*,*N*-dimethylformamidal (DMF) reported earlier by Wudl and coworkers afforded a different fullerene product, C_60_H(CN) [16]. Another example was the mechanochemical reaction of C_60_ and *N*-benzhydryl sulfonamides in the presence of FeCl_3_ to give fulleroindanes, which could not be obtained in ODCB or 1,1,2,2-tetrachloroethane (TCE) solution at 120 °C [17]. A more recent example reported by Bolm, Hernández, and coworkers was the reaction of aldehydes with amines and CaC_2_ under ball-milling conditions to give 1,4-diamino-2-butynes [18]. In contrast, terminal propargylamines were the major products by heating the same reaction mixtures in undried acetonitrile [19]. The use of CaC_2_, a solid replacement of hazardous and difficult-to-handle acetylene gas, in organic synthesis has been widely reported [20,21,22,23,24]. Nevertheless, its application is still limited because of its very poor solubility in organic solvents. Moreover, an Oxone (2KHSO_5_·KHSO_4_·K_2_SO_4_)-NaCl-Na_2_CO_3_ system could react with aldoximes to afford nitrile oxides [25]. In this context, we attempted the reaction of CaC_2_ with (*E*)-4-methylbenzaldehyde oxime (**1a**), NaCl, Oxone, and Na_2_CO_3_ in order to obtain 3,4-di-*p*-tolyl-3*a*,6*a*-dihydroisoxazolo[4,5-*d*]isoxazole via double [2 + 3] cycloadditions between the in situ generated acetylene and 1,3-dipolar nitrile oxide under ball-milling conditions. Unexpectedly, (*Z*)-4-methyl-*N*-((4-methylbenzoyl)oxy)benzimidoyl chloride (**2a**) was obtained as the major product with no participation of CaC_2_ (Scheme 1). To further investigate this unexpected reaction pathway, we conducted the present work.

## 2. Results

At the outset of our study, **1a** was selected as the representative substrate to screen the optimal reaction conditions. When a mixture of **1a** (0.2 mmol), NaCl (2 equiv.), CaC_2_ (1 equiv.), Oxone (2 equiv.), and Na_2_CO_3_ (2 equiv.) along with four stainless steel balls (5 mm in diameter) were introduced into a stainless steel jar (5 mL) and milled vigorously at a frequency of 30 Hz in a Retsch MM400 mixer mill (Retsch GmbH, Haan, Germany) at room temperature for 30 min, an unexpected product **2a** was isolated in 23% yield (Table 1, entry 1). The structure of product **2a** was unambiguously established by single-crystal X-ray diffraction analysis (see the Supplementary Materials for details). Because CaC_2_ did not take part in the formation of **2a**, the same reaction was repeated without CaC_2_. Similar results, that is, 24% yield for **2a**, were obtained (entry 2). Encouraged by this initial result, the employed oxidant and base, molar ratio of the reactants, reaction time, as well as the liquid-assisted grinding (LAG) agent [26], were screened to attain the optimal conditions, and the results are shown in Table 1. At first, we tried to replace Oxone with K_2_S_2_O_8_, Na_2_S_2_O_8_, (NH_4_)_2_S_2_O_8_, 1,4-benzoquinone quinone (BQ), and PhI(OAc)_2_, but all of these reactions failed and indicated that Oxone was the best oxidant (entries 3–7). Then, the replacement of Na_2_CO_3_ with other different bases, including NaHCO_3_, NaOAc, NaO*^t^*Bu, KO*^t^*Bu, Cs_2_CO_3_, 1,8-diazabicyclo[5.4.0]undec-7-ene (DBU), 4-dimethylaminopyridine (DMAP), and 1,4-diazabicyclo[2.2.2]octane (DABCO), gave inferior results (entries 8–15). Subsequently, the amounts of the reactants were varied. The yield of **2a** was only 7% if 1 equiv. of Oxone was used (entry 16), while a better yield of 41% was obtained by using 3 equiv. of Oxone (entry 17). To our delight, the yield of **2a** was increased to 57% when the amount of Oxone was further increased to 4 equiv. (entry 18). Further screening the amounts of Na_2_CO_3_ and NaCl revealed that the best molar ratio of **1a**, NaCl, Oxone, and Na_2_CO_3_ was still 1:2:4:2 (entries 19–23 vs. entry 18). Based on these results, the effect of the reaction time on the product yield was investigated. However, the yield by either shortening to 20 min or prolonging to 40 min was not improved (entries 24 and 25 vs. entry 18). The LAG protocol has been known as a powerful tool to promote mechanochemical reaction [1,13,14,26]. Therefore, several liquids including dichloromethane (DCM), EtOH, and H_2_O were added to the reaction mixture as LAG agents. Unfortunately, the yield of **2a** could not be improved (entries 26–28). Therefore, the optimal reaction conditions were determined as follows: 0.2 mmol of **1a**, 2 equiv. of NaCl, 4 equiv. of Oxone, and 2 equiv. of Na_2_CO_3_ at 30 Hz for 30 min under solvent-free ball-milling conditions (entry 18).

**Scheme 2:**
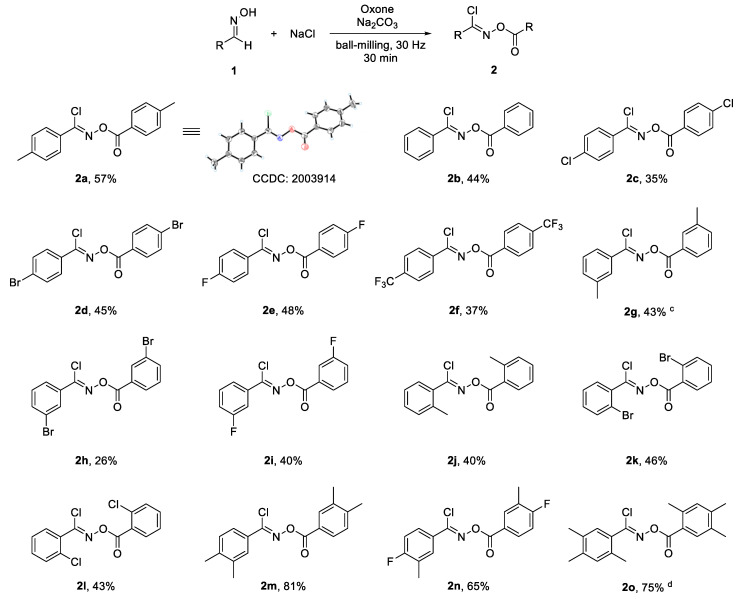
The reaction of aldoximes with NaCl and Oxone under ball-milling conditions ^a,b^. ^a^ Unless otherwise noted, the reactions were performed with **1** (0.2 mmol), NaCl (0.4 mmol), Oxone (0.8 mmol), and Na_2_CO_3_ (0.4 mmol) together with four stainless steel balls (5 mm in diameter) in a stainless steel jar (5 mL). ^b^ Isolated yields based on **1**. ^c^ NaCl (0.6 mmol), Na_2_CO_3_ (0.2 mmol). ^d^ Na_2_CO_3_ (0.8 mmol).

With the optimized reaction conditions in hand, the substrate scope and functional group tolerance of this reaction were then investigated with a range of aldoximes **1a**–**o** (Scheme 2). In general, benzaldehyde oximes **1a**–**o** with both electron-donating and electron-withdrawing groups on the phenyl ring were tolerated under our reaction conditions, affording the corresponding products **2a**–**o** in moderate to good yields.

Firstly, the substrate **1b** with no substituent was examined and afforded **2b** in 44% yield. The *para*-halogen-substituted (*E*)-benzaldehyde oximes **1c**–**e** also worked and afforded the desired products **2c**–**e** in 35–48% yields. For the substrate **1f** with the *para*-substituted CF_3_ group, the corresponding product **2f** could be isolated in 37% yield. As for the *meta*-substituted substrates **1g**–**i** bearing Me, Br, and F, the desired products **2g**–**i** were synthesized in 26–43% yields. Substrates **1j**–**l** with the *ortho*-substituted Me, Br, and Cl groups were also compatible, resulting in products **2j**–**l** in 40–46% yields. Delightedly, the disubstituted substrates **1m** and **1n** performed well, furnishing products **2m** and **2n** in 81% and 65% yields, respectively. The trisubstituted substrate **1o** also underwent well to generate the desired product **2o** in a good yield of 75%. Taking the multistep processes from the aldoximes to *N*-acyloxyimidoyl chlorides (vide infra) into account, the yields of ~40–60% in most cases were satisfactory.

**Scheme 3:**
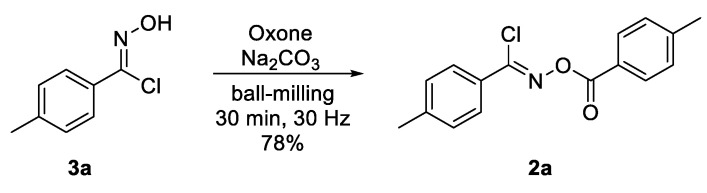
The reaction of **3a** with Oxone and Na_2_CO_3_ under ball-milling conditions.

It was believed that hydroximoyl chloride **3** might be the key intermediate of this reaction. To prove our assumption, control experiments were performed. The reaction of (*Z*)-*N*-hydroxy-4-methylbenzimidoyl chloride (**3a**) (0.2 mmol) with Na_2_CO_3_ (1 equiv.) and Oxone (2 equiv.) under solvent-free ball-milling conditions indeed afforded **2a** in 78% yield (Scheme 3). However, **2a** could not be formed from **3a** in the presence of just Na_2_CO_3_ under the same ball-milling conditions, indicating that Oxone was essential for the mechanochemical transformation of **3a** to **2a**.

**Scheme 4:**
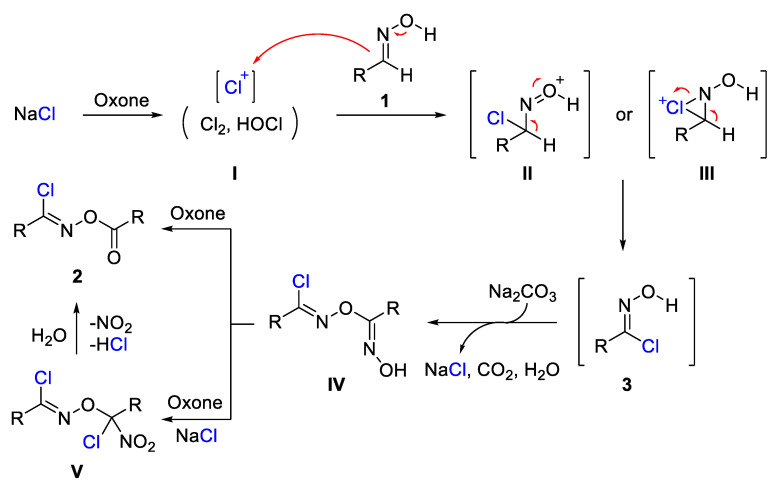
A plausible reaction pathway.

Although the exact reaction mechanism towards the formation of **2** is not clear, a proposed reaction pathway is shown in Scheme 4 based on the aforementioned experiments and previous literature [25,27,28]. First, NaCl is oxidized to the chlorinating species **I** in the presence of Oxone [25]. Next, **I** undergoes chlorination reaction with **1** to generate the hydroximoyl chloride **3** via intermediate **II** or **III** [25]. The key precursor **3** is then dimerized to **IV** in the presence of Na_2_CO_3_. Subsequently, the deoximation of **IV** by Oxone [27] and/or a combination of excess Oxone and NaCl via the *gem*-chloronitro intermediate **V** and following hydrolysis [28] provides the final product **2**.

## 3. Discussion

The formation of **2b** as a byproduct in 12% yield upon distillation of the chlorination product of benzaldoxime in methylene chloride was previously reported by Chiang [29]. Subsequently, the same author disclosed that the thermolysis of *N*-hydroxybenzimidoyl chloride (**3b**) at 180 °C (8 mm) afforded 70% of phenyl isocyanate and 21% of **2b** [30]. Similarly, product **2c** was also isolated in 10% yield along with 4-chlorobenzonitrile from the pyrolysis of 4-chloro-*N*-hydroxybenzimidoyl chloride (**3c**) [30]. These product yields are lower than those from aldoximes (see Scheme 2) and should be much lower than those from the same hydroximoyl chlorides under our mechanochemical conditions (see Scheme 3).

The oxidation reactions of aldoximes have been widely investigated in organic synthesis, among which the most typical one is to be oxidized to 1,3-dipolar nitrile oxide, which undergoes cyclization reaction with alkenes or alkynes to form isoxazolines [25]. When it comes to the α-chlorination of aldoximes, *N*-chlorosuccinimide (NCS) [31] is the most common chlorinating agent, chlorine (Cl_2_) [29,30] is also used, yet it is hard to handle. Compared to the other reagents utilized for the α-chlorination of aldoximes, including benzyltrimethylammonium tetrachloroiodate (BTMA ICl_4_) [32] and *t*-butyl hypochlorite (*t*-BuOCl) [33], NaCl-Oxone is more convenient, cheaper and easier to handle. In the present work, when aldoximes were treated with NaCl, Oxone, and Na_2_CO_3_ under our solvent-free ball-milling conditions, *N*-acyloxyimidoyl chlorides were obtained unexpectedly. However, when NaCl was replaced with NCS, NaBr, or NaI, neither the corresponding *N*-acyloxyimidoyl halides, nor hydroximoyl halides could be isolated except for some yet-unidentified products.

**Scheme 5:**
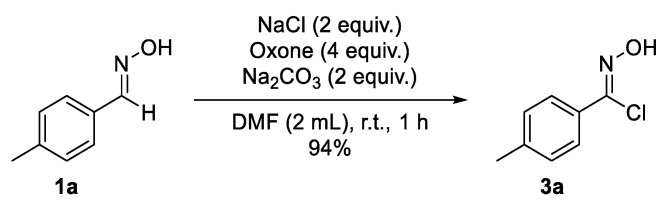
The reaction of **1a** with NaCl, Oxone, and Na_2_CO_3_ in DMF.

For comparison purpose, the present reaction was also carried out under liquid-phase conditions. By using the reaction of **1a** with NaCl, Oxone, and Na_2_CO_3_ as an example, a number of solvents and different reaction temperature were examined, hydroximoyl chloride **3a** instead of **2a** was obtained in all cases. Our best result is shown in Scheme 5. When a mixture of **1a** (0.2 mmol), NaCl (2 equiv.), Oxone (4 equiv.), and Na_2_CO_3_ (2 equiv.) was stirred in *N*,*N*-dimethylformamide (DMF) at room temperature for 1 h, **3a** was obtained in 94% yield. It is obvious that different products, **2a** and **3a**, are formed under solvent-free mechanochemical conditions and liquid-phase conditions. The most likely reason is that the possibility for the close contact of two neighboring molecules of **3a** to dimerize under solvent-free conditions is much higher than that under liquid-phase conditions [15]. This result provided another example to showcase the uniqueness of altering the reaction pathway by the mechanochemical protocol [15,17,18]. It should be noted that the expected product shown in Scheme 1 still could not be obtained from the reaction of **1a** with CaC_2_, NaCl, Oxone, and Na_2_CO_3_; compound **3a** was obtained in essentially the same yield as that without CaC_2_ after stirring for 1 h (Scheme 1), while a similar complex mixture with no obvious predominant product was observed for the reaction with or without CaC_2_ after 24 h. These results indicated that CaC_2_ did not participate in the present liquid-phase reaction, and a longer reaction time could not afford **2a**, **3a**, or any other meaningful product.

## 4. Experimental Section

### 4.1. General Information

All reagents were obtained from commercial sources and used without further purification. NMR spectra were recorded on a Bruker Advance III HD 400 NMR spectrometer (Bruker BioSpin AG, Fällanden, Switzerland; 400 MHz for ^1^H NMR; 101 MHz for ^13^C NMR; 376 MHz for ^19^F NMR) and a Bruker Advance III HD 500 NMR spectrometer (Bruker BioSpin AG, Fällanden, Switzerland; 500 MHz for ^1^H NMR; 126 MHz for ^13^C NMR; 471 MHz for ^19^F NMR). ^1^H NMR chemical shifts were determined relative to CDCl_3_ at *δ* 7.26 ppm. ^13^C NMR chemical shifts were determined relative to CDCl_3_ at *δ* 77.16 ppm. Data are reported as follows: chemical shift (*δ*, ppm), multiplicity (s = singlet, d = doublet, t = triplet, m = multiplet, q = quartet). High-resolution mass spectra (HRMS) were taken on a Waters Acquity UPLC-Xevo G2 QTof mass spectrometer (Waters, Milford, MA, USA) with FTMS-ESI in positive mode. Ball-milling reactions were performed in a MM400 mixer mill (Retsch GmbH, Haan, Germany), using a 5 mL stainless steel jar with four 5 mm diameter stainless steel balls and were milled vigorously at a frequency of 1800 rounds per minute (30 Hz) at room temperature. Aldoximes **1** were prepared according to the reported protocol [25]. Single crystals of **2a** were grown from dichloromethane/*n*-hexane at 4 °C.

### 4.2. Synthesis and Characterization of Products 2

A mixture of **1** (0.2 mmol, 1.0 equiv.), NaCl (0.4 mmol, 2 equiv.), Oxone (0.8 mmol, 4.0 equiv.), and Na_2_CO_3_ (0.4 mmol, 2.0 equiv.) together with four stainless balls (5 mm in diameter) were introduced into a stainless steel jar (5 mL). The reaction vessel and another identical vessel were closed and fixed on the vibration arms of a Retsch MM400 mixer mill, and were vibrated vigorously at a rate of 1800 rounds per minute (30 Hz) at room temperature for 30 min. After completion of the reaction, the resulting mixtures from the two runs were combined and extracted with dichloromethane and water. The organic layer was decanted, and the aqueous layer was extracted by dichloromethane (2 × 20 mL). The combined organic extracts were evaporated to remove the solvent in vacuo. The residue was separated by flash column chromatography on silica gel with ethyl acetate/petroleum ether as the eluent to afford **2**.

*(Z)-4-Methyl-N-((4-methylbenzoyl)oxy)benzimidoyl chloride (***2a***)*. By following the general procedure, the reaction of **1a** (55.6 mg, 0.4 mmol) with NaCl (48.0 mg, 0.8 mmol), Oxone (987.3 mg, 1.6 mmol), and Na_2_CO_3_ (85.5 mg, 0.8 mmol) afforded **2a** (32.7 mg, 57% yield). White solid; ^1^H NMR (400 MHz, CDCl_3_) *δ* 8.08 (d, *J* = 8.1 Hz, 2H), 7.93 (d, *J* = 8.1 Hz, 2H), 7.29 (d, *J* = 8.1 Hz, 2H), 7.25 (d, *J* = 8.1 Hz, 2H), 2.43 (s, 3H), 2.40 (s, 3H); ^13^C NMR (101 MHz, CDCl_3_) *δ* 162.9, 147.9, 144.8, 142.8, 130.2 (2C), 129.5 (2C), 129.4 (2C), 128.7, 128.3 (2C), 125.3, 21.9, 21.6; HRMS (FTMS-ESI) Calcd for C_16_H_14_^35^ClNO_2_Na [M + Na]^+^ 310.0605; found 310.0613.

*(Z)-N-(Benzoyloxy)benzimidoyl chloride* (**2b**). By following the general procedure, the reaction of **1b** (44 μL, 0.4 mmol) with NaCl (48.5 mg, 0.8 mmol), Oxone (993.5 mg, 1.6 mmol) and Na_2_CO_3_ (86.8 mg, 0.8 mmol) afforded **2b** (22.8 mg, 44% yield). White solid; ^1^H NMR (400 MHz, CDCl_3_) *δ* 8.21 (d, *J* = 7.5 Hz, 2H), 8.06 (d, *J* = 7.5 Hz, 2H), 7.65 (t, *J* = 7.4 Hz, 1H), 7.57–7.43 (m, 5H); ^13^C NMR (101 MHz, CDCl_3_) *δ* 162.8, 148.1, 134.0, 132.3, 131.5, 130.2 (2C), 128.82 (2C), 128.76 (2C), 128.4 (2C), 128.1; HRMS (FTMS-ESI) Calcd for C_14_H_10_^35^ClNO_2_Na [M + Na]^+^ 282.0292; found 282.0299.

*(Z)-4-Chloro-N-((4-chlorobenzoyl)oxy)benzimidoyl chloride* (**2c**). By following the general procedure, the reaction of **1c** (62.8 mg, 0.4 mmol) with NaCl (47.2 mg, 0.8 mmol), Oxone (983.6 mg, 1.6 mmol) and Na_2_CO_3_ (85.4 mg, 0.8 mmol) afforded **2c** (22.9 mg, 35% yield). White solid; ^1^H NMR (500 MHz, CDCl_3_) *δ* 8.12 (d, *J* = 8.6 Hz, 2H), 7.98 (d, *J* = 8.7 Hz, 2H), 7.49 (d, *J* = 8.6 Hz, 2H), 7.44 (d, *J* = 8.7 Hz, 2H); ^13^C NMR (126 MHz, CDCl_3_) *δ* 161.8, 147.3, 140.7, 138.8, 131.5 (2C), 129.8, 129.6 (2C), 129.3 (2C), 129.1 (2C), 126.3; HRMS (FTMS-ESI) Calcd for C_14_H_9_^35^Cl_3_NO_2_ [M + H]^+^ 327.9693; found 327.9685.

*(Z)-4-Bromo-N-((4-bromobenzoyl)oxy)benzimidoyl chloride* (**2d**). By following the general procedure, the reaction of **1d** (80.7 mg, 0.4 mmol) with NaCl (48.0 mg, 0.8 mmol), Oxone (984.9 mg, 1.6 mmol) and Na_2_CO_3_ (86.3 mg, 0.8 mmol) afforded **2d** (37.2 mg, 45% yield). White solid; ^1^H NMR (500 MHz, CDCl_3_) *δ* 8.04 (d, *J* = 8.5 Hz, 2H), 7.92 (d, *J* = 8.6 Hz, 2H), 7.67 (d, *J* = 8.5 Hz, 2H), 7.61 (d, *J* = 8.6 Hz, 2H); ^13^C NMR (126 MHz, CDCl_3_) *δ* 162.0, 147.5, 132.3 (2C), 132.1 (2C), 131.6 (2C), 130.3, 129.8 (2C), 129.5, 127.3, 126.8; HRMS (FTMS-ESI) Calcd for C_14_H_9_^79^Br_2_^35^ClNO_2_ [M + H]^+^ 415.8683; found 415.8683.

*(Z)-4-Fluoro-N-((4-fluorobenzoyl)oxy)benzimidoyl chloride* (**2e**). By following the general procedure, the reaction of **1e** (57.2 mg, 0.4 mmol) with NaCl (47.3 mg, 0.8 mmol), Oxone (985.0 mg, 1.6 mmol) and Na_2_CO_3_ (86.3 mg, 0.8 mmol) afforded **2e** (28.6 mg, 48% yield). White solid; ^1^H NMR (400 MHz, CDCl_3_) *δ* 8.21 (dd, *J* = 8.9, 5.4 Hz, 2H), 8.06 (dd, *J* = 9.0, 5.2 Hz, 2H), 7.24–7.12 (m, 4H); ^13^C NMR (101 MHz, CDCl_3_) *δ* 166.4 (d, *J*_C-F_ = 255.9 Hz), 165.31 (d, *J*_C-F_ = 254.2 Hz), 161.8, 147.2, 132.8 (d, *J*_C-F_ = 9.5 Hz, 2C), 130.7 (d, *J*_C-F_ = 9.0 Hz, 2C), 127.5 (d, *J*_C-F_ = 3.3 Hz), 124.2 (d, *J*_C-F_ = 3.0 Hz), 116.2 (d, *J*_C-F_ = 22.2 Hz, 2C), 116.1 (d, *J*_C-F_ = 22.2 Hz, 2C); ^19^F NMR (376 MHz, CDCl_3_) *δ* –103.35 to –103.43 (m, 1F), –106.51 to –106.59 (m, 1F); HRMS (FTMS-ESI) Calcd for C_14_H_9_^35^ClF_2_NO_2_ [M + H]^+^ 296.0284; found 296.0289.

*(Z)-4-(Trifluoromethyl)-N-((4-(trifluoromethyl)benzoyl)oxy)benzimidoyl chloride* (**2f**). By following the general procedure, the reaction of **1f** (76.3 mg, 0.4 mmol) with NaCl (48.8 mg, 0.8 mmol), Oxone (987.0 mg, 1.6 mmol) and Na_2_CO_3_ (85.3 mg, 0.8 mmol) afforded **2f** (29.5 mg, 37% yield). White solid; ^1^H NMR (500 MHz, CDCl_3_) *δ* 8.32 (d, *J* = 8.2 Hz, 2H), 8.06 (d, *J* = 8.2 Hz, 2H), 7.81 (d, *J* = 8.2 Hz, 2H), 7.75 (d, *J* = 8.2 Hz, 2H); ^13^C NMR (126 MHz, CDCl_3_) *δ* 161.4, 147.5, 135.6 (q, *J*_C-F_ = 32.9 Hz), 134.6, 134.1 (q, *J*_C-F_ = 32.9 Hz), 131.1, 130.7 (2C), 128.9 (2C), 126.0 (q, *J*_C-F_ = 3.8 Hz, 2C), 125.9 (q, *J*_C-F_ = 3.7 Hz, 2C), 123.5 (q, *J*_C-F_ = 272.6 Hz), 123.4 (q, *J*_C-F_ = 272.9 Hz); ^19^F NMR (471 MHz, CDCl_3_) *δ* –63.09 (s, 3F), –63.26 (s, 3F); HRMS (FTMS-ESI) Calcd for C_16_H_8_^35^ClF_6_NO_2_Na [M + Na]^+^ 418.0040; found 418.0060.

*(Z)-3-Methyl-N-((3-methylbenzoyl)oxy)benzimidoyl chloride* (**2g**). By following the general procedure, the reaction of **1g** (55.5 mg, 0.4 mmol) with NaCl (71.1 mg, 1.2 mmol), Oxone (985.7 mg, 1.6 mmol) and Na_2_CO_3_ (43.2 mg, 0.4 mmol) afforded **2g** (24.7 mg, 43% yield). White solid; ^1^H NMR (500 MHz, CDCl_3_) *δ* 8.03–7.99 (m, 2H), 7.88 (s, 1H), 7.85–7.81 (m, 1H), 7.45 (d, *J* = 7.6 Hz, 1H), 7.40 (t, *J* = 7.9 Hz, 1H), 7.37–7.33 (m, 2H); ^13^C NMR (126 MHz, CDCl_3_) *δ* 163.1, 148.3, 138.7, 138.6, 134.8, 133.1, 131.4, 130.7, 128.8, 128.71, 128.65, 128.0, 127.3, 125.8, 21.5, 21.4; HRMS (FTMS-ESI) Calcd for C_16_H_15_^35^ClNO_2_ [M + H]^+^ 288.0786; found 288.0787.

*(Z)-3-Bromo-N-((3-bromobenzoyl)oxy)benzimidoyl chloride* (**2h**). By following the general procedure, the reaction of **1h** (88.4 mg, 0.4 mmol) with NaCl (48.6 mg, 0.8 mmol), Oxone (987.0 mg, 1.6 mmol) and Na_2_CO_3_ (85.8 mg, 0.4 mmol) afforded **2h** (22.0 mg, 26% yield). White solid; ^1^H NMR (400 MHz, CDCl_3_) *δ* 8.31 (s, 1H), 8.19 (s, 1H), 8.13 (d, *J* = 7.9 Hz, 1H), 7.99 (d, *J* = 7.9 Hz, 1H), 7.79 (d, *J* = 7.9 Hz, 1H), 7.68 (d, *J* = 7.9 Hz, 1H), 7.41 (t, *J* = 7.9 Hz, 1H), 7.35 (t, *J* = 7.9 Hz, 1H); ^13^C NMR (101 MHz, CDCl_3_) *δ* 161.4, 147.2, 137.1, 135.4, 133.2, 133.1, 131.2, 130.5, 130.3, 129.8, 128.8, 127.1, 122.97, 122.95; HRMS (FTMS-ESI) Calcd for C_14_H_8_^79^Br_2_^35^ClNO_2_Na [M + Na]^+^ 437.8503; found 437.8503.

*(Z)-3-Fluoro-N-((3-fluorobenzoyl)oxy)benzimidoyl chloride* (**2i**). By following the general procedure, the reaction of **1i** (59.3 mg, 0.4 mmol) with NaCl (47.3 mg, 0.8 mmol), Oxone (986.0 mg, 1.6 mmol) and Na_2_CO_3_ (85.8 mg, 0.8 mmol) afforded **2i** (23.6 mg, 40% yield). White solid; ^1^H NMR (400 MHz, CDCl_3_) *δ* 8.00 (d, *J* = 7.8 Hz, 1H), 7.90–7.84 (m, 2H), 7.77 (dt, *J* = 9.5, 1.9 Hz, 1H), 7.52 (td, *J* = 8.0, 5.6 Hz, 1H), 7.46 (td, *J* = 8.1, 5.8 Hz, 1H), 7.36 (td, *J* = 8.2, 2.2 Hz, 1H), 7.29–7.22 (m, 1H); ^13^C NMR (101 MHz, CDCl_3_) *δ* 162.74 (d, *J*_C-F_ = 248.1 Hz), 162.71 (d, *J*_C-F_ = 247.6 Hz), 161.6 (d, *J*_C-F_ = 3.2 Hz), 147.3 (d, *J*_C-F_ = 3.3 Hz), 133.4 (d, *J*_C-F_ = 8.4 Hz), 130.6 (d, *J*_C-F_ = 7.8 Hz), 130.5 (d, *J*_C-F_ = 8.1 Hz), 130.0 (d, *J*_C-F_ = 7.5 Hz), 126.0 (d, *J*_C-F_ = 3.2 Hz), 124.2 (d, *J*_C-F_ = 3.1 Hz), 121.3 (d, *J*_C-F_ = 21.3 Hz), 119.5 (d, *J*_C-F_ = 21.2 Hz), 117.1 (d, *J*_C-F_ = 23.5 Hz), 115.6 (d, *J*_C-F_ = 24.7 Hz); ^19^F NMR (376 MHz, CDCl_3_) *δ* –111.26 to –111.35 (m, 1F), –111.56 to –111.65 (m, 1F); HRMS (FTMS-ESI) Calcd for C_14_H_9_^35^ClF_2_NO_2_ [M + H]^+^ 296.0284; found 296.0291.

*(Z)-2-Methyl-N-((2-methylbenzoyl)oxy)benzimidoyl chloride* (**2j**). By following the general procedure, the reaction of **1j** (59.1 mg, 0.4 mmol) with NaCl (48.3 mg, 0.8 mmol), Oxone (987.6 mg, 1.6 mmol) and Na_2_CO_3_ (86.3 mg, 0.8 mmol) afforded **2j** (22.8 mg, 40% yield). White solid; ^1^H NMR (400 MHz, CDCl_3_) *δ* 8.12 (d, *J* = 7.6 Hz, 1H), 7.59 (d, *J* = 7.4 Hz, 1H), 7.49 (t, *J* = 7.4 Hz, 1H), 7.42–7.25 (m, 5H), 2.71 (s, 3H), 2.56 (s, 3H); ^13^C NMR (101 MHz, CDCl_3_) *δ* 163.4, 147.2, 141.5, 137.5, 133.1, 132.2, 132.1, 131.2, 131.1 (2C), 130.0, 127.2, 126.1 (2C), 22.0, 20.7; HRMS (FTMS-ESI) Calcd for C_16_H_14_^35^ClNO_2_Na [M + Na]^+^ 310.0605; found 310.0612.

*(Z)-2-Bromo-N-((2-bromobenzoyl)oxy)benzimidoyl chloride* (**2k**). By following the general procedure, the reaction of **1k** (81.7 mg, 0.4 mmol) with NaCl (49.3 mg, 0.8 mmol), Oxone (985.0 mg, 1.6 mmol) and Na_2_CO_3_ (87.7 mg, 0.8 mmol) afforded **2k** (38.5 mg, 46% yield). White solid; ^1^H NMR (500 MHz, CDCl_3_) *δ* 7.98 (dd, *J* = 7.3, 2.1 Hz, 1H), 7.74 (dd, *J* = 7.6, 1.4 Hz, 1H), 7.67 (d, *J* = 7.9 Hz, 1H), 7.55 (dd, *J* = 7.6, 1.6 Hz, 1H), 7.47–7.39 (m, 3H), 7.36 (td, *J* = 7.7, 1.6 Hz, 1H); ^13^C NMR (126 MHz, CDCl_3_) *δ* 162.0, 147.2, 134.8, 133.9, 133.69, 133.67, 132.4, 132.0, 131.3, 129.6, 127.6, 127.5, 122.6, 122.1; HRMS (FTMS-ESI) Calcd for C_14_H_8_^79^Br_2_^35^ClNO_2_Na [M + Na]^+^ 437.8503; found 437.8511.

*(Z)-2-Chloro-N-((2-chlorobenzoyl)oxy)benzimidoyl chloride* (**2l**). By following the general procedure, the reaction of **1l** (64.4 mg, 0.4 mmol) with NaCl (50.3 mg, 0.8 mmol), Oxone (986.0 mg, 1.6 mmol) and Na_2_CO_3_ (85.9 mg, 0.8 mmol) afforded **2l** (28.5 mg, 43% yield). White solid; ^1^H NMR (400 MHz, CDCl_3_) *δ* 8.02 (d, *J* = 8.0 Hz, 1H), 7.60–7.33 (m, 7H); ^13^C NMR (101 MHz, CDCl_3_) *δ* 161.6, 146.3, 134.7, 133.7, 133.4, 132.3, 132.1, 131.9, 131.5, 131.3, 130.6, 127.7, 127.1, 126.9; HRMS (FTMS-ESI) Calcd for C_14_H_8_^35^Cl_3_NO_2_Na [M + Na]^+^ 349.9513; found 349.9519.

*(Z)-N-((3,4-Dimethylbenzoyl)oxy)-3,4-dimethylbenzimidoyl chloride* (**2m**). By following the general procedure, the reaction of **1m** (62.6 mg, 0.4 mmol) with NaCl (51.1 mg, 0.4 mmol), Oxone (986.1 mg, 1.6 mmol) and Na_2_CO_3_ (86.9 mg, 0.8 mmol) afforded **2m** (50.9 mg, 81% yield). White solid; ^1^H NMR (400 MHz, CDCl_3_) *δ* 7.95 (s, 1H), 7.94 (d, *J* = 8.0 Hz, 1H), 7.83 (s, 1H), 7.76 (d, *J* = 7.8 Hz, 1H), 7.26 (d, *J* = 7.8 Hz, 1H), 7.21 (d, *J* = 8.0 Hz, 1H), 2.35 (s, 6H), 2.32 (s, 6H); ^13^C NMR (101 MHz, CDCl_3_) *δ* 163.2, 148.1, 143.6, 141.6, 137.3, 137.2, 131.2, 130.1, 130.0, 129.2, 129.1, 127.7, 126.1, 125.6, 20.3, 20.0, 19.9, 19.8; HRMS (FTMS-ESI) Calcd for C_18_H_18_^35^ClNO_2_Na [M + Na]^+^ 338.0918; found 338.0922.

*(Z)-4-Fluoro-N-((4-fluoro-3-methylbenzoyl)oxy)-3-methylbenzimidoyl chloride* (**2n**). By following the general procedure, the reaction of **1n** (63.9 mg, 0.4 mmol) with NaCl (48.4 mg, 0.8 mmol), Oxone (986.8 mg, 1.6 mmol) and Na_2_CO_3_ (86.9 mg, 0.8 mmol) afforded **2n** (41.8 mg, 65% yield). White solid; ^1^H NMR (500 MHz, CDCl_3_) *δ* 8.07–8.00 (m, 2H), 7.91 (d, *J* = 7.1 Hz, 1H), 7.87–7.82 (m, 1H), 7.15–7.05 (m, 2H), 2.36 (s, 3H), 2.33 (s, 3H); ^13^C NMR (126 MHz, CDCl_3_) *δ* 165.0 (d, *J*_C-F_ = 254.5 Hz), 163.9 (d, *J*_C-F_ = 252.7 Hz), 162.1, 147.3, 133.9 (d, *J*_C-F_ = 6.7 Hz), 131.7 (d, *J*_C-F_ = 6.1 Hz), 130.1 (d, *J*_C-F_ = 9.5 Hz), 128.1 (d, *J*_C-F_ = 9.0 Hz), 127.2 (d, *J*_C-F_ = 3.5 Hz), 125.9 (d, *J*_C-F_ = 18.1 Hz), 125.8 (d, *J*_C-F_ = 18.1 Hz), 123.8 (d, *J*_C-F_ = 3.3 Hz), 115.7 (d, *J*_C-F_ = 23.3 Hz), 115.6 (d, *J*_C-F_ = 23.3 Hz), 14.67 (d, *J*_C-F_ = 4.8 Hz), 14.65 (d, *J*_C-F_ = 4.7 Hz); ^19^F NMR (471 MHz, CDCl_3_) *δ* –107.66 to –107.74 (m, 1F), –110.77 to –110.85 (m, 1F); HRMS (FTMS-ESI) Calcd for C_16_H_12_^35^ClF_2_NO_2_Na [M + Na]^+^ 346.0417; found 346.0421.

*(Z)-2,4,5-Trimethyl-N-((2,4,5-trimethylbenzoyl)oxy)benzimidoyl chloride* (**2o**). By following the general procedure, the reaction of **1o** (67.2 mg, 0.4 mmol) with NaCl (53.5 mg, 0.8 mmol), Oxone (996.0 mg, 1.6 mmol) and Na_2_CO_3_ (178.1 mg, 1.6 mmol) afforded **2o** (51.5 mg, 75% yield). White solid; ^1^H NMR (500 MHz, CDCl_3_) *δ* 7.88 (s, 1H), 7.36 (s, 1H), 7.08 (s, 1H), 7.05 (s, 1H), 2.63 (s, 3H), 2.48 (s, 3H), 2.30 (s, 6H), 2.27 (s, 3H), 2.26 (s, 3H); ^13^C NMR (126 MHz, CDCl_3_) *δ* 163.7, 147.2, 142.5, 140.0, 138.9, 134.7, 134.3, 133.5, 132.5, 132.1, 131.0 (2C), 129.6, 124.4, 21.6, 20.3, 20.0, 19.8, 19.4, 19.3; HRMS (FTMS-ESI) Calcd for C_20_H_22_^35^ClNO_2_Na [M + Na]^+^ 366.1231; found 366.1239.

### 4.3. Liquid-Phase Synthesis and Characterization of 3a

To a stirred solution of **2a** (0.2 mmol) in DMF (2 mL) were added NaCl (23.8 mg, 0.4 mmol), Na_2_CO_3_ (42.6 mg, 0.4 mmol) and Oxone (493.1 mg, 0.8 mmol). The reaction mixture was allowed to stir at room temperature for 1 h. Then, the reaction mixture was filtered through a silica gel plug with ethyl acetate as the eluent, and subsequently the solvent was removed under reduced pressure. The residue was purified by flash chromatography on silica gel with petroleum ether/ethyl acetate as eluent to give product **3a** (31.9 mg, 94% yield).

*(Z)-N-Hydroxy-4-methylbenzimidoyl chloride* (**3a**). Pale yellow solid; ^1^H NMR (500 MHz, CDCl_3_) *δ* 8.43 (s, 1H), 7.72 (d, *J* = 8.2 Hz, 2H), 7.21 (d, *J* = 8.2 Hz, 2H), 2.39 (s, 3H); ^13^C NMR (126 MHz, CDCl_3_) δ 141.3, 140.5, 129.8, 129.4 (2C), 127.3 (2C), 21.5; HRMS (FTMS-ESI) Calcd for C_8_H_9_^35^ClNO [M + H]^+^ 170.0367; found 170.0367.

### 4.4. Mechanochemical Synthesis of 2a from 3a

A mixture of **3a** (0.1 mmol, 17.2 mg), Oxone (0.2 mmol, 125.4 mg), and Na_2_CO_3_ (0.1 mmol, 11.1 mg) together with four stainless balls (5 mm in diameter) were introduced into a stainless steel jar (5 mL). The reaction vessel and another same vessel were closed and fixed on the vibration arms of a Retsch MM400 mixer mill, and were vibrated vigorously at a rate of 1800 rounds per minute (30 Hz) at room temperature for 30 min. After completion of the reaction, the resulting mixtures from the two runs were combined and extracted with dichloromethane and water. The organic layer was decanted, and the aqueous layer was extracted by dichloromethane (2 × 20 mL). The combined organic extracts were evaporated to remove the solvent in vacuo. The residue was separated by flash column chromatography on silica gel with ethyl acetate/petroleum ether as the eluent to afford **2a** (22.4 mg, 78% yield).

## 5. Conclusions

In summary, we have disclosed the unexpected formation of *N*-acyloxyimidoyl chlorides from the solvent-free reaction of aldoximes with NaCl and Oxone under ball-milling conditions, while hydroximoyl chlorides are obtained from the liquid-phase counterparts. The present work provides another verification that mechanochemistry can alter the reaction pathways leading to different products compared to the solution-based counterparts.

## Figures and Tables

**Table 1:**
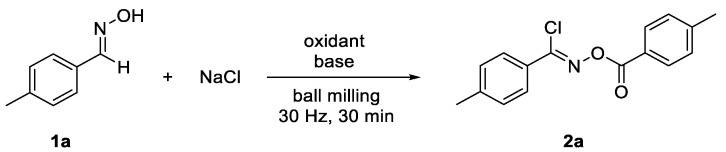
Optimization of the reaction conditions ^a^.

Entry	NaCl (equiv.)	Oxidant (equiv.)	Base (equiv.)	Yield of 2a (%) ^b^
1	2.0	Oxone (2.0)	Na_2_CO_3_ (2.0)	23 ^c^
2	2.0	Oxone (2.0)	Na_2_CO_3_ (2.0)	24
3	2.0	K_2_S_2_O_8_ (2.0)	Na_2_CO_3_ (2.0)	trace
4	2.0	Na_2_S_2_O_8_ (2.0)	Na_2_CO_3_ (2.0)	trace
5	2.0	(NH_4_)_2_S_2_O_8_ (2.0)	Na_2_CO_3_ (2.0)	0
6	2.0	BQ (2.0)	Na_2_CO_3_ (2.0)	trace
7	2.0	PhI(OAc)_2_ (2.0)	Na_2_CO_3_ (2.0)	trace
8	2.0	Oxone (2.0)	NaHCO_3_ (2.0)	10
9	2.0	Oxone (2.0)	NaOAc (2.0)	6
10	2.0	Oxone (2.0)	NaO*^t^*Bu (2.0)	12
11	2.0	Oxone (2.0)	KO*^t^*Bu (2.0)	14
12	2.0	Oxone (2.0)	Cs_2_CO_3_ (2.0)	9
13	2.0	Oxone (2.0)	DBU (2.0)	0
14	2.0	Oxone (2.0)	DMAP (2.0)	0
15	2.0	Oxone (2.0)	DABCO (2.0)	0
16	2.0	Oxone (1.0)	Na_2_CO_3_ (2.0)	7
17	2.0	Oxone (3.0)	Na_2_CO_3_ (2.0)	41
**18**	**2.0**	**Oxone (4.0)**	**Na_2_CO_3_ (2.0)**	**57**
19	2.0	Oxone (5.0)	Na_2_CO_3_ (2.0)	46
20	2.0	Oxone (4.0)	Na_2_CO_3_ (1.0)	35
21	2.0	Oxone (4.0)	Na_2_CO_3_ (3.0)	29
22	1.0	Oxone (4.0)	Na_2_CO_3_ (2.0)	39
23	3.0	Oxone (4.0)	Na_2_CO_3_ (2.0)	43
24 ^d^	2.0	Oxone (4.0)	Na_2_CO_3_ (2.0)	48
25 ^e^	2.0	Oxone (4.0)	Na_2_CO_3_ (2.0)	37
26 ^f,g^	2.0	Oxone (4.0)	Na_2_CO_3_ (2.0)	40
27 ^f,h^	2.0	Oxone (4.0)	Na_2_CO_3_ (2.0)	trace
28 ^f,i^	2.0	Oxone (4.0)	Na_2_CO_3_ (2.0)	45

^a^ Unless otherwise noted, the reactions were carried out in a Retsch MM400 mixer mill with 0.2 mmol of **1a**. ^b^ Isolated yields based on **1a**. ^c^ CaC_2_ (1 equiv.) was added. ^d^ 20 min. ^e^ 40 min. ^f^ A liquid (100 µL, *η* = 0.17 µL/mg) was added as a LAG agent. ^g^ DCM was added. ^h^ EtOH was added. ^i^ H_2_O was added.

## Supplementary Materials

The following are available online, ^1^H and ^13^C NMR spectra of **2**, **3a,** X-ray structure and crystal data of **2a**.
