# Supplementary material for: Reaction of Aldoximes with Sodium Chloride and Oxone under Ball-Milling Conditions
Source: Molecules. 2020 Aug 14;25(16):3719. doi: 10.3390/molecules25163719 (PMC7463692; doi:10.3390/molecules25163719)
Supplement: Supplementary file 1 [file molecules-25-03719-s001.pdf]

molecules

Supplementary Materials

Reaction of Aldoximes with Sodium Chloride and Oxone under Ball-Milling Conditions

Kuan Chen [1], Chuang Niu [1] and Guan-Wu Wang [1,2,*]

[1] Hefei National Laboratory for Physical Sciences at Microscale and Department of Chemistry, University of Science and Technology of China, Hefei, Anhui 230026, P. R. China; kuanc@mail.ustc.edu.cn (K.C.); cniu@mail.ustc.edu.cn (C.N.)
[2] State Key Laboratory of Applied Organic Chemistry, Lanzhou University, Lanzhou, Gansu 730000, P. R. China
* Correspondence: gwang@ustc.edu.cn; Tel: +86-551-6360-7864

Table of contents

1. **^{1}H and ^{13}C NMR spectra of 2 and 3a** 2
2. **Single-Crystal X-ray Crystallography of 2a** 20

1. ¹H and ¹³C NMR spectra of 2 and 3a

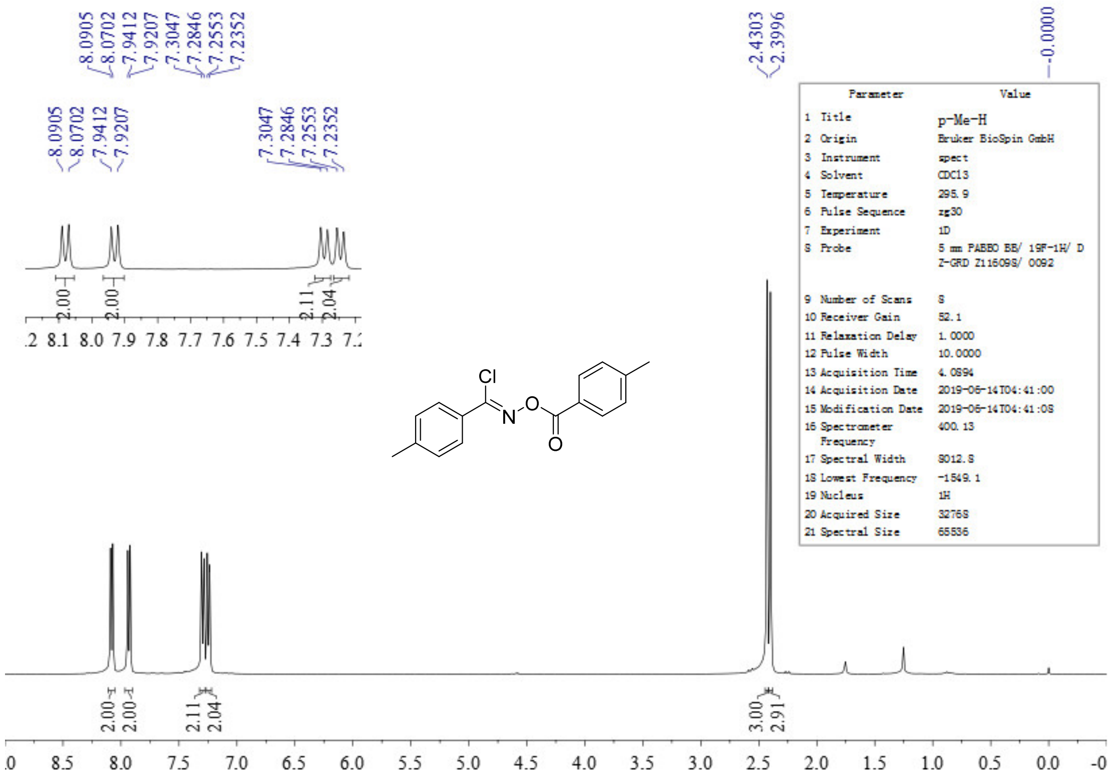

Figure S1. ¹H NMR (400 MHz, $CDCl_3$) spectrum of compound 2a.

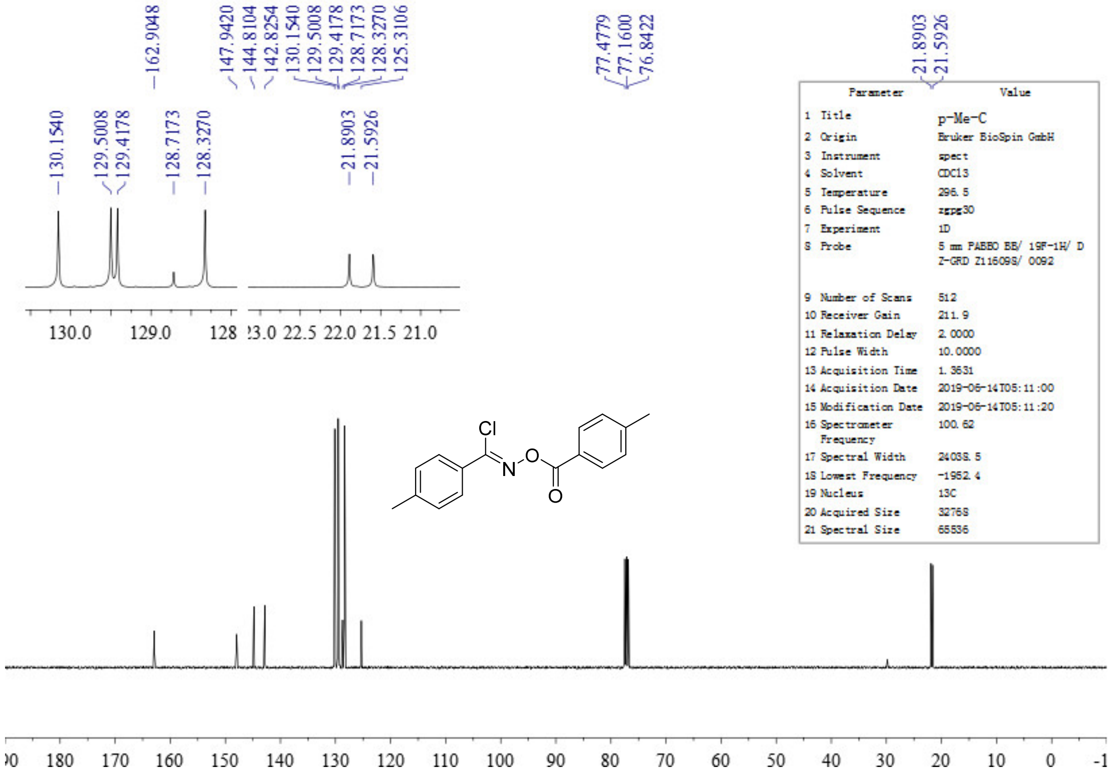

Figure S2. ¹³C NMR (101 MHz, $CDCl_3$) spectrum of compound 2a.

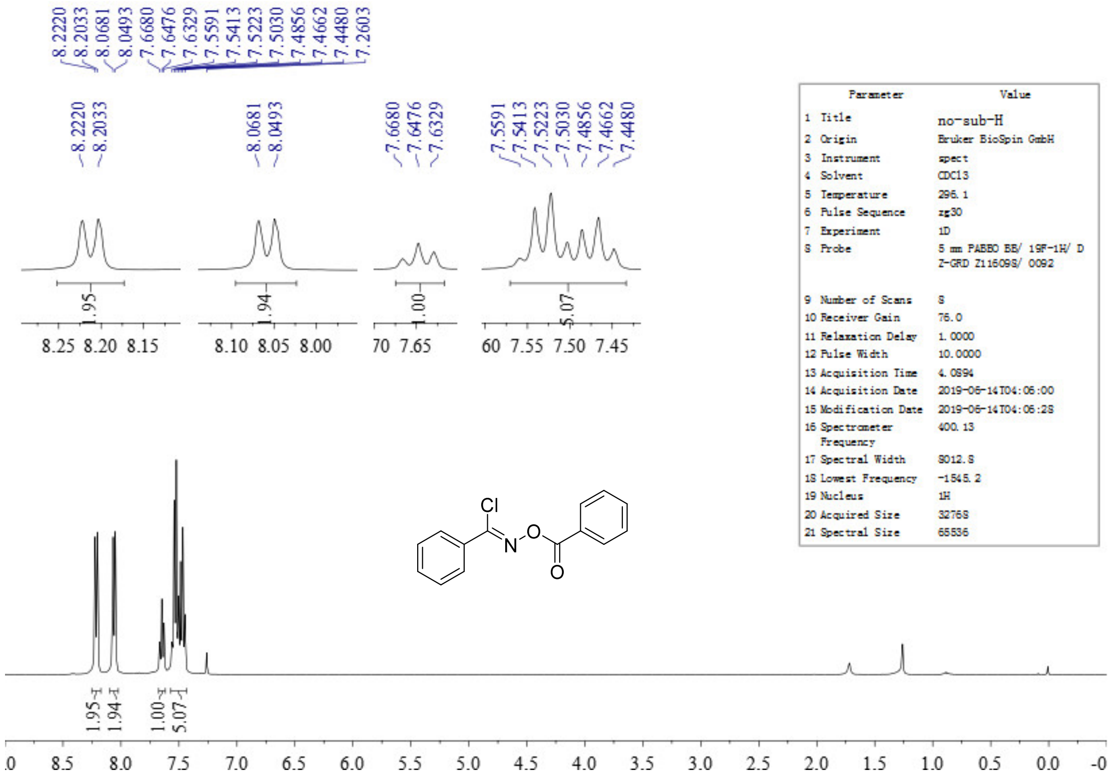

Figure S3. ^{1}H NMR (400 MHz, $CDCl_3$) spectrum of compound 2b.

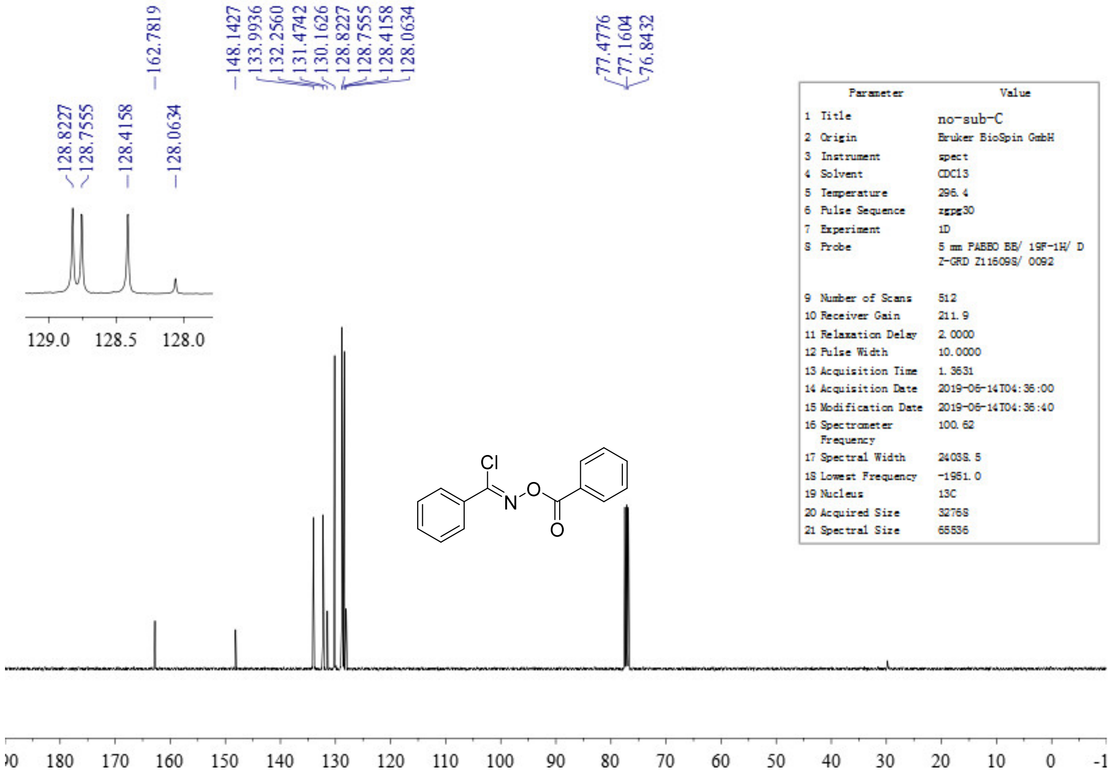

Figure S4. ^{13}C NMR (101 MHz, $CDCl_3$) spectrum of compound 2b.

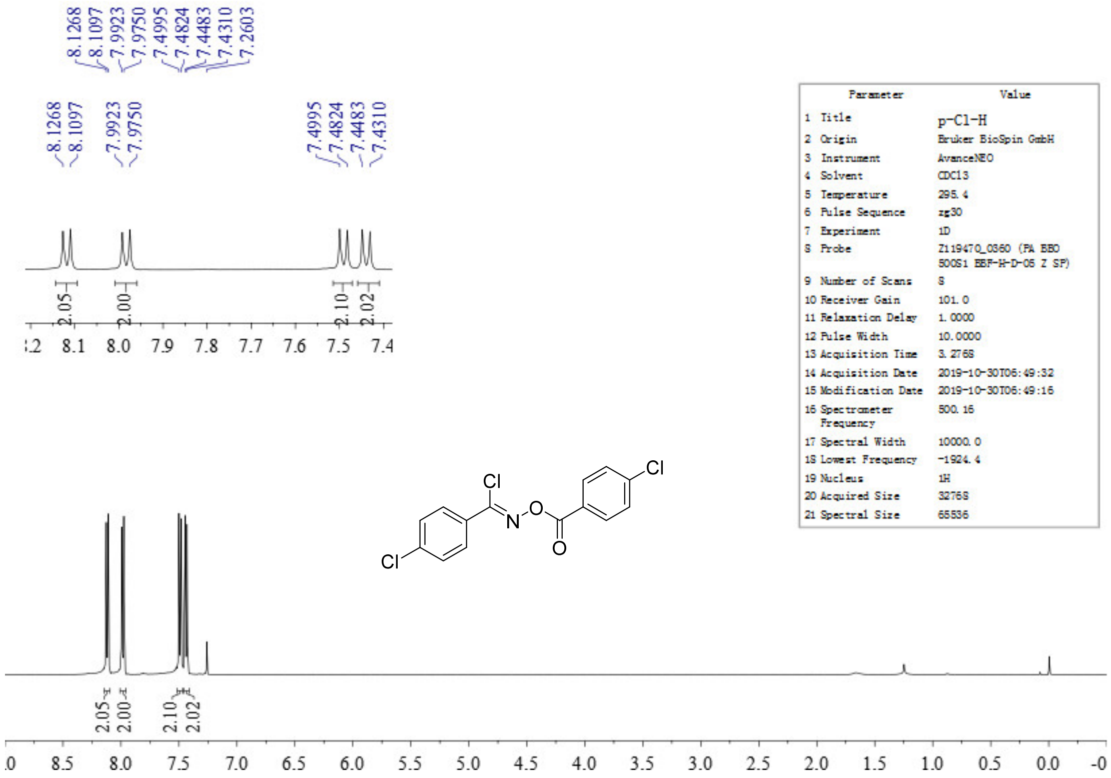

Figure S5. ^1H NMR (500 MHz, $CDCl_3$) spectrum of compound 2c.

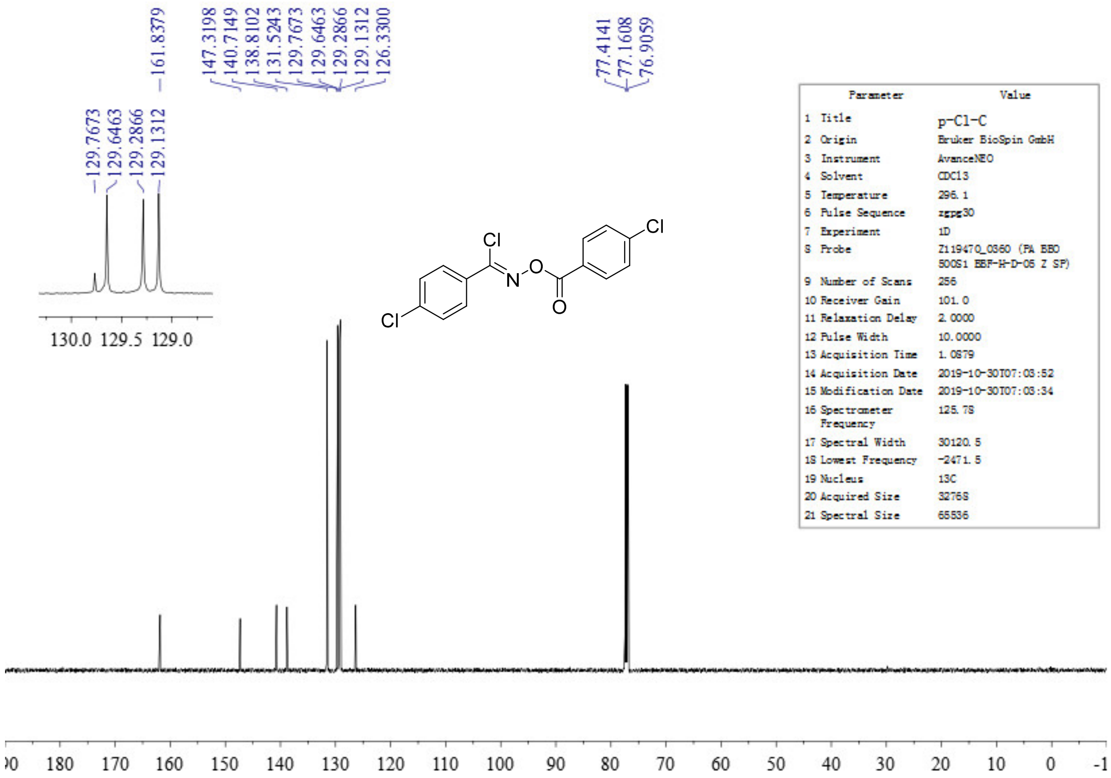

Figure S6. ^{13}C NMR (126 MHz, $CDCl_3$) spectrum of compound 2c.

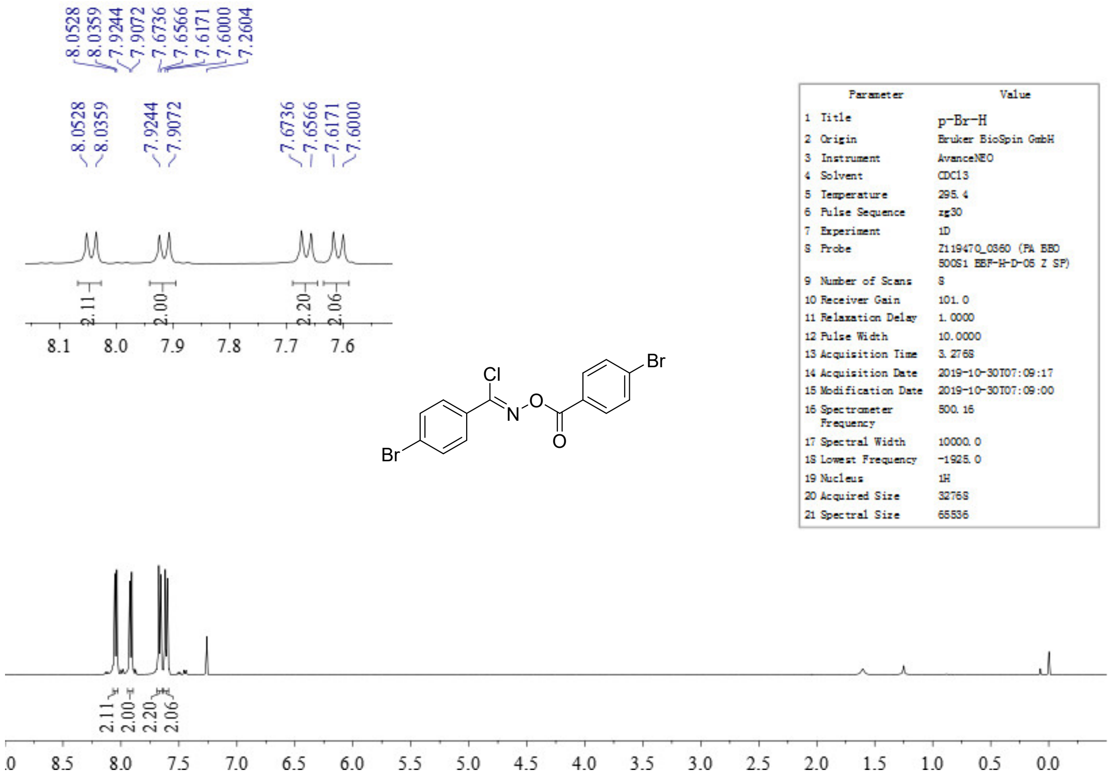

Figure S7. ^{1}H NMR (500 MHz, $CDCl_3$) spectrum of compound 2d.

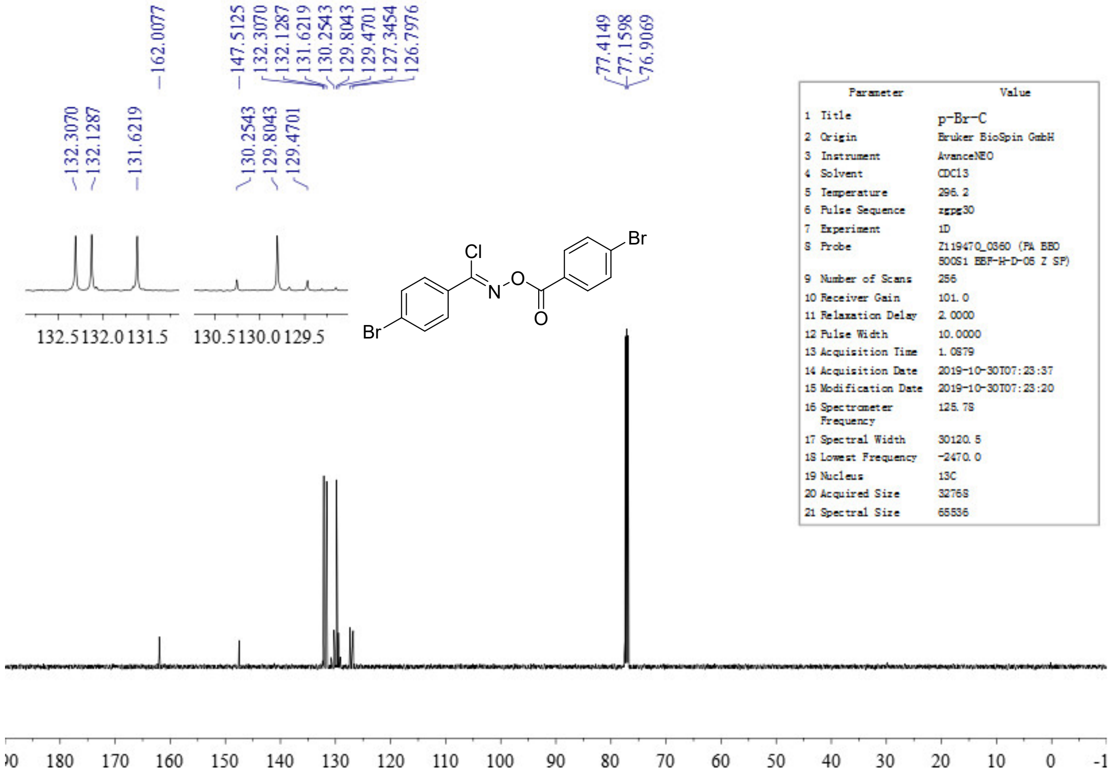

Figure S8. ^{13}C NMR (126 MHz, $CDCl_3$) spectrum of compound 2d.

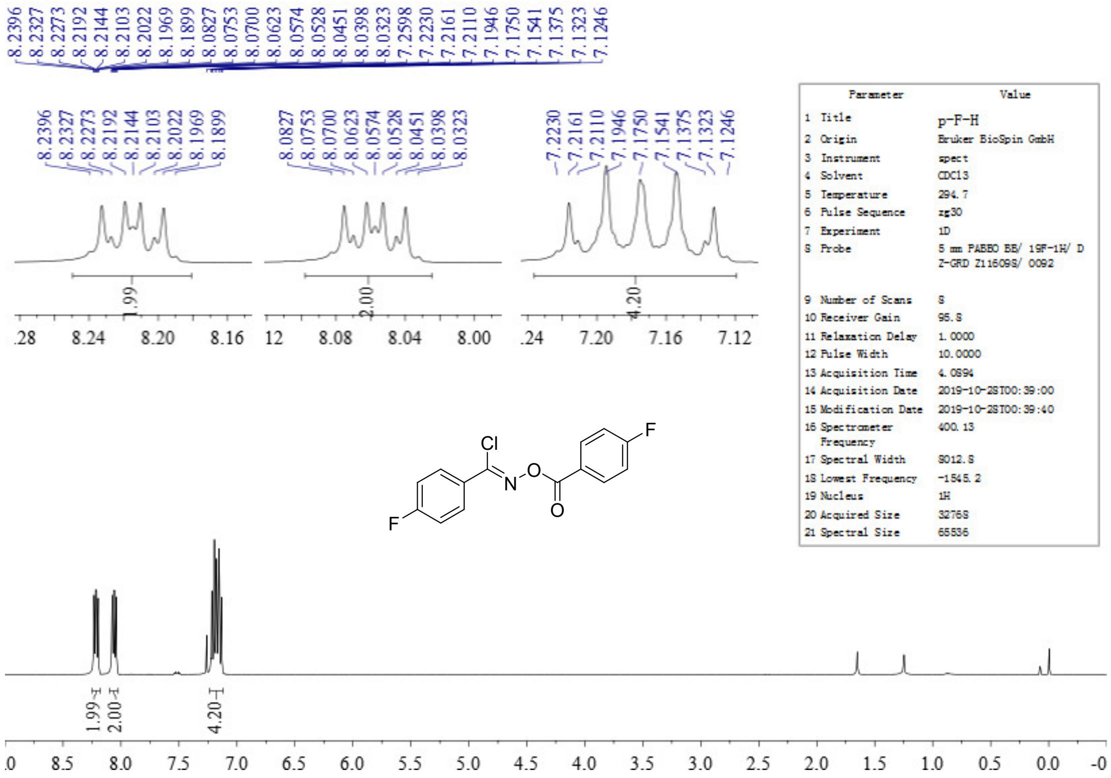

Figure S9. ^1H NMR (400 MHz, $CDCl_3$) spectrum of compound 2e.

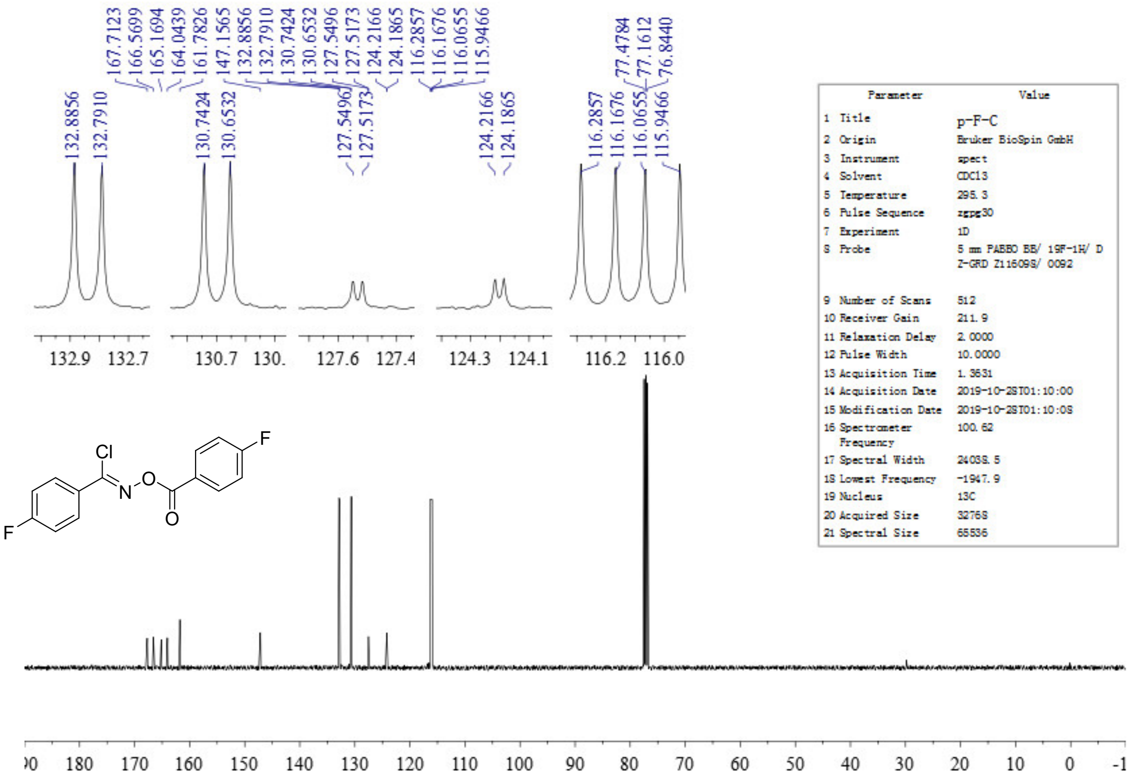

Figure S10. ^{13}C NMR (101 MHz, $CDCl_3$) spectrum of compound 2e.

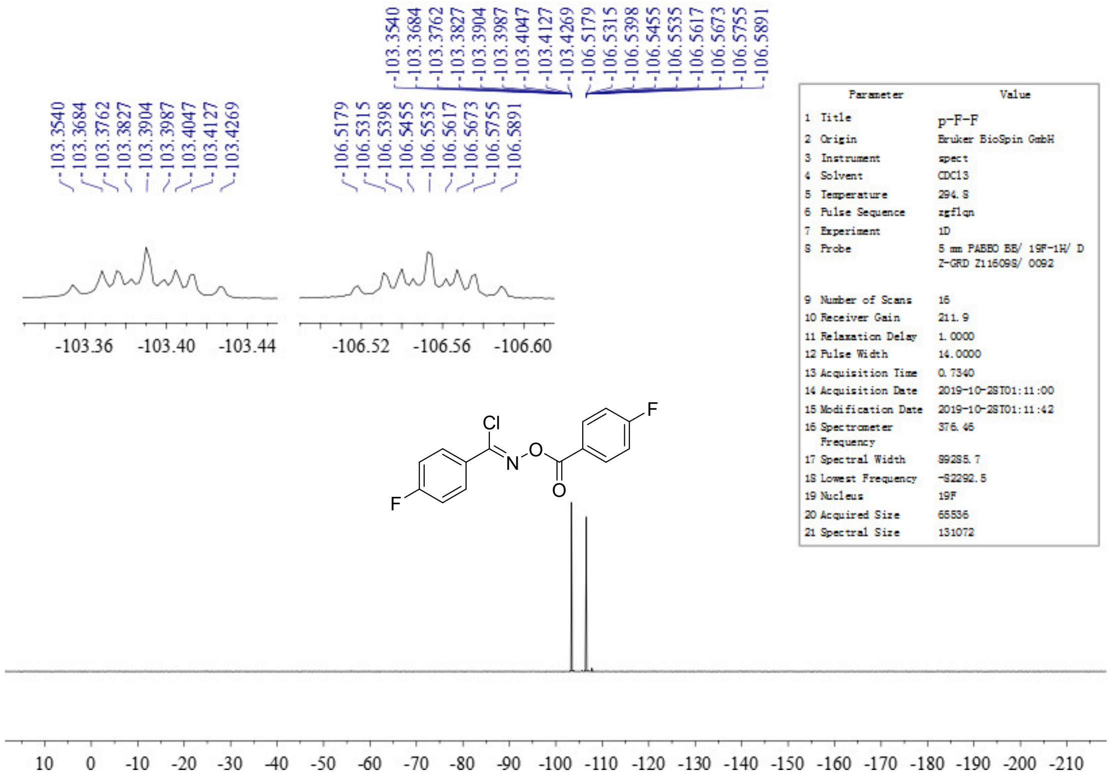

Figure S11. ^{19}F NMR (376 MHz, $CDCl_3$) spectrum of compound 2e.

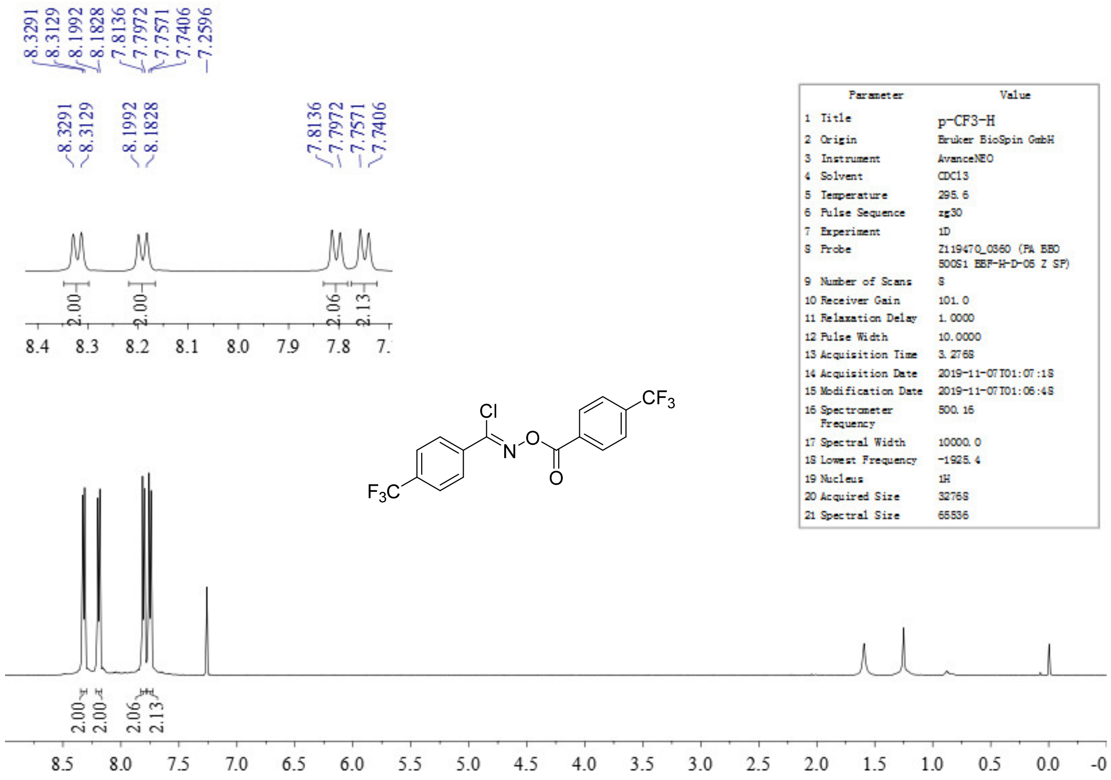

Figure S12. ^{1}H NMR (500 MHz, $CDCl_3$) spectrum of compound 2f.

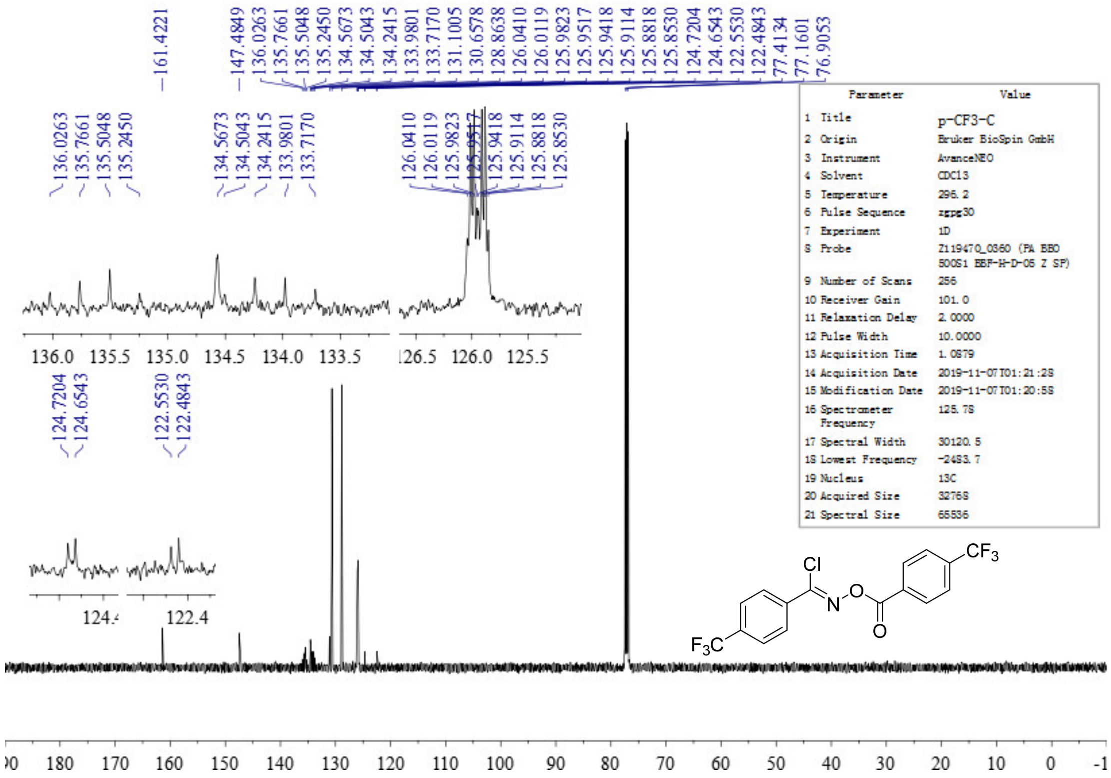

Figure S13. ^{13}C NMR (126 MHz, $CDCl_3$) spectrum of compound 2f.

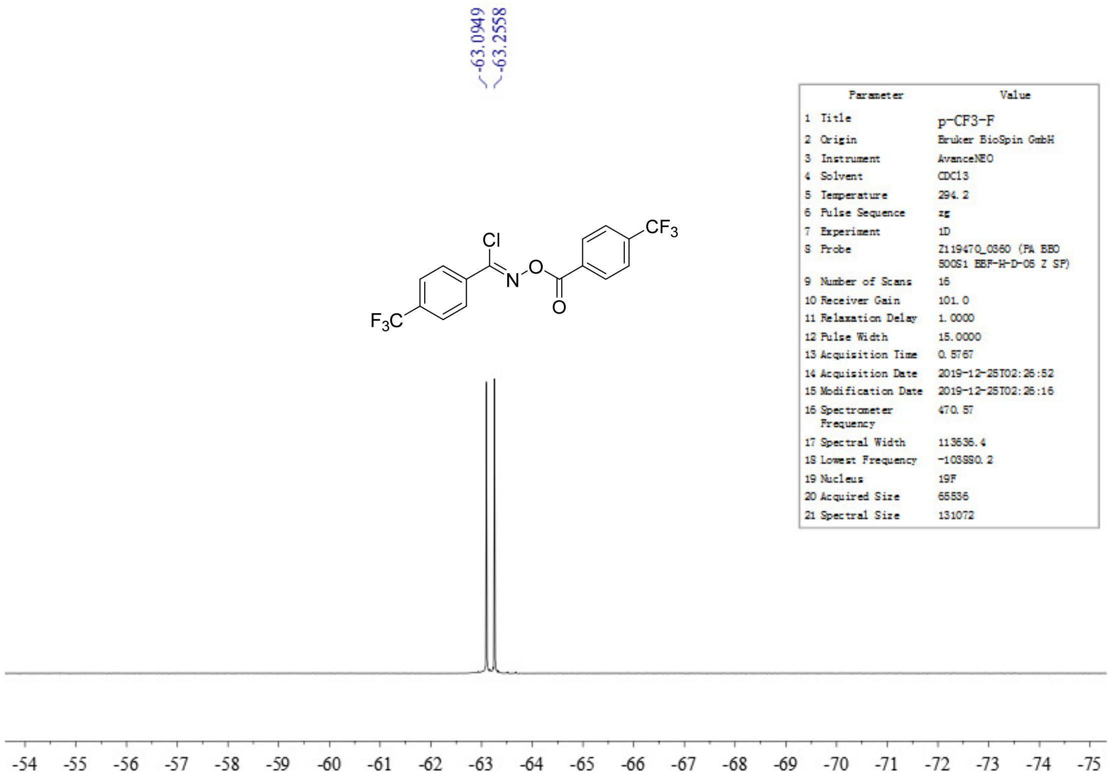

Figure S14. ^{19}F NMR (471 MHz, $CDCl_3$) spectrum of compound 2f.

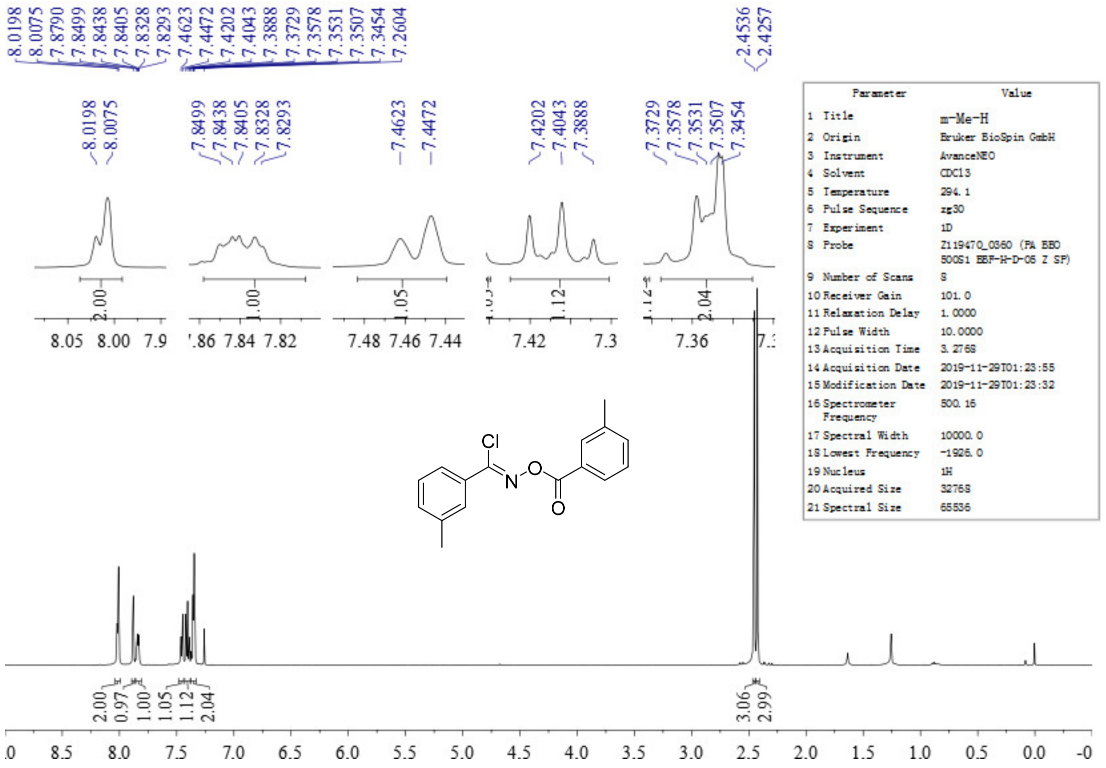

Figure S15. ^{1}H NMR (500 MHz, $CDCl_3$) spectrum of compound 2g.

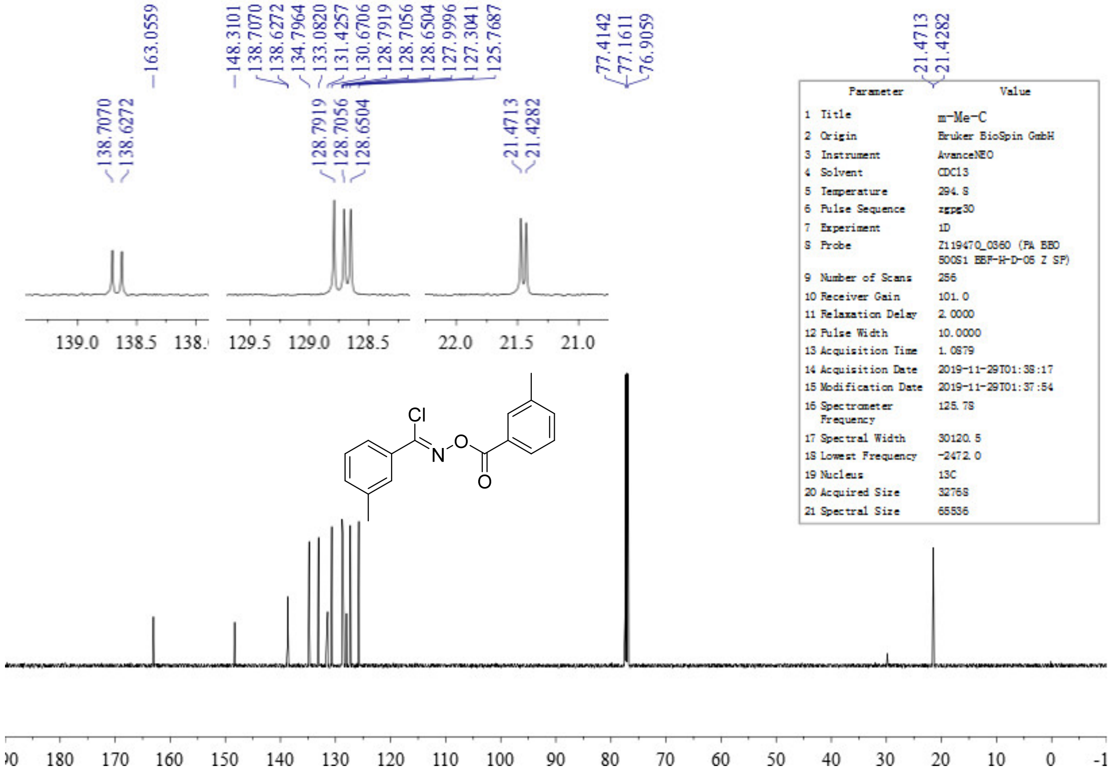

Figure S16. ^{13}C NMR (126 MHz, $CDCl_3$) spectrum of compound 2g.

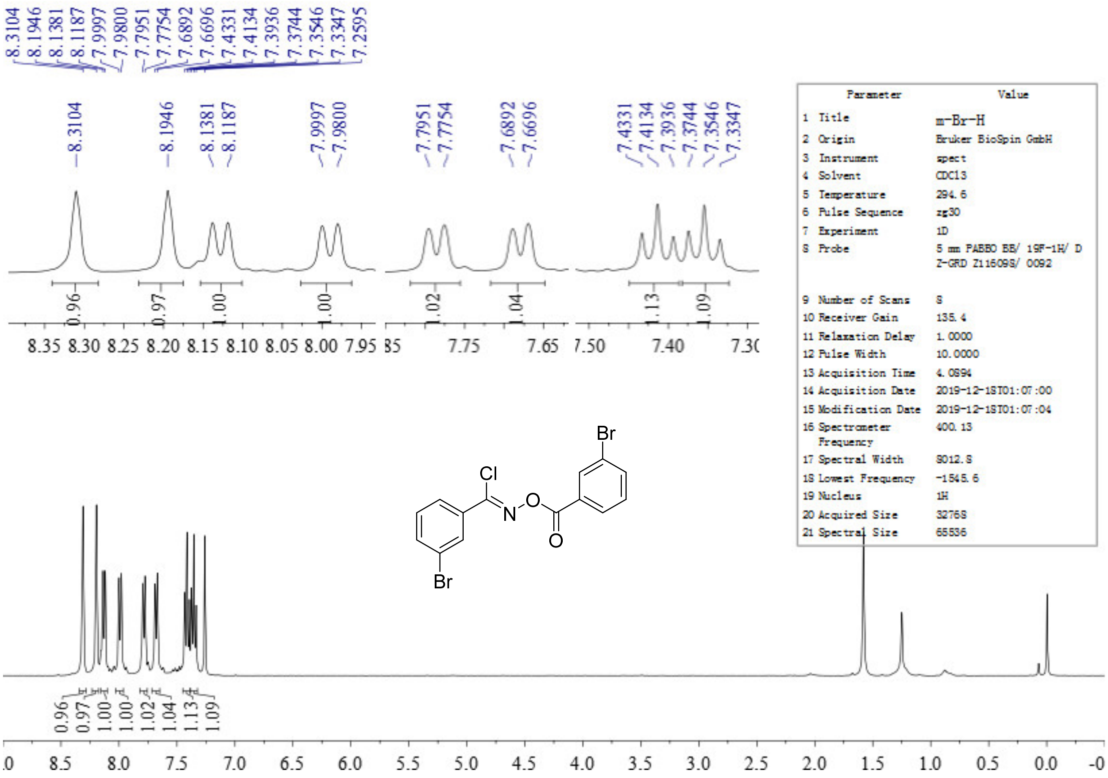

Figure S17. ^{1}H NMR (400 MHz, $CDCl_3$) spectrum of compound 2h.

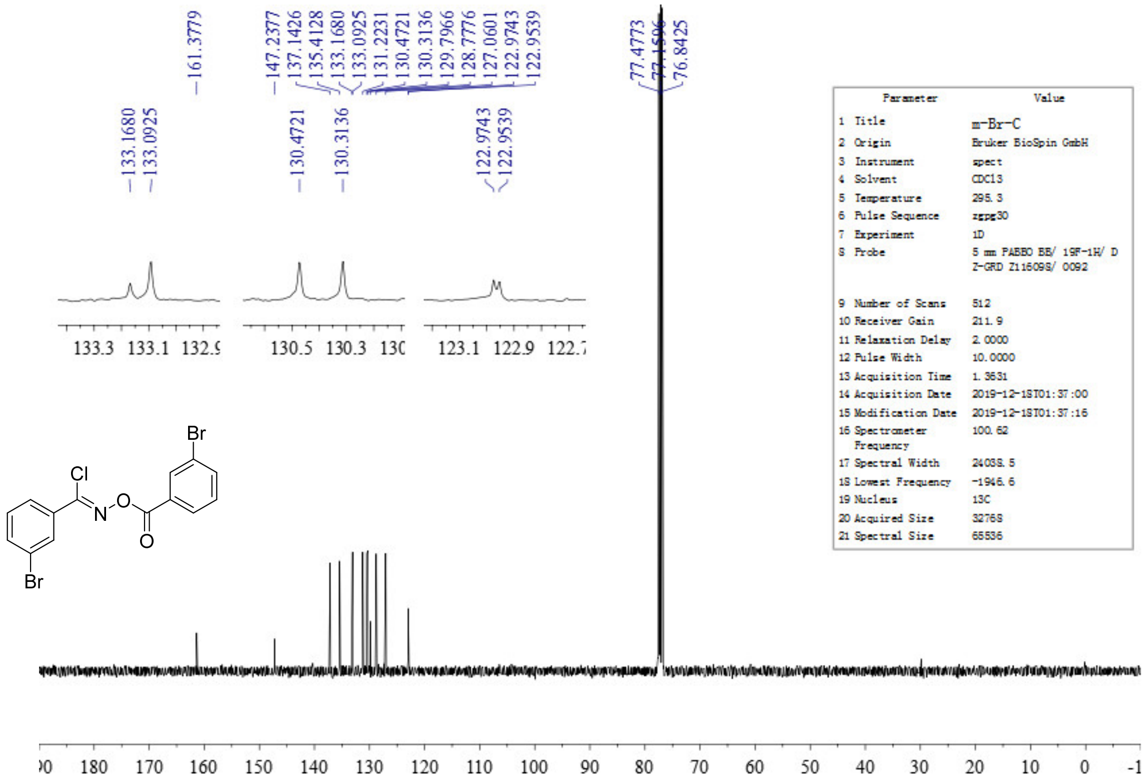

Figure S18. ^{13}C NMR (101 MHz, $CDCl_3$) spectrum of compound 2h.

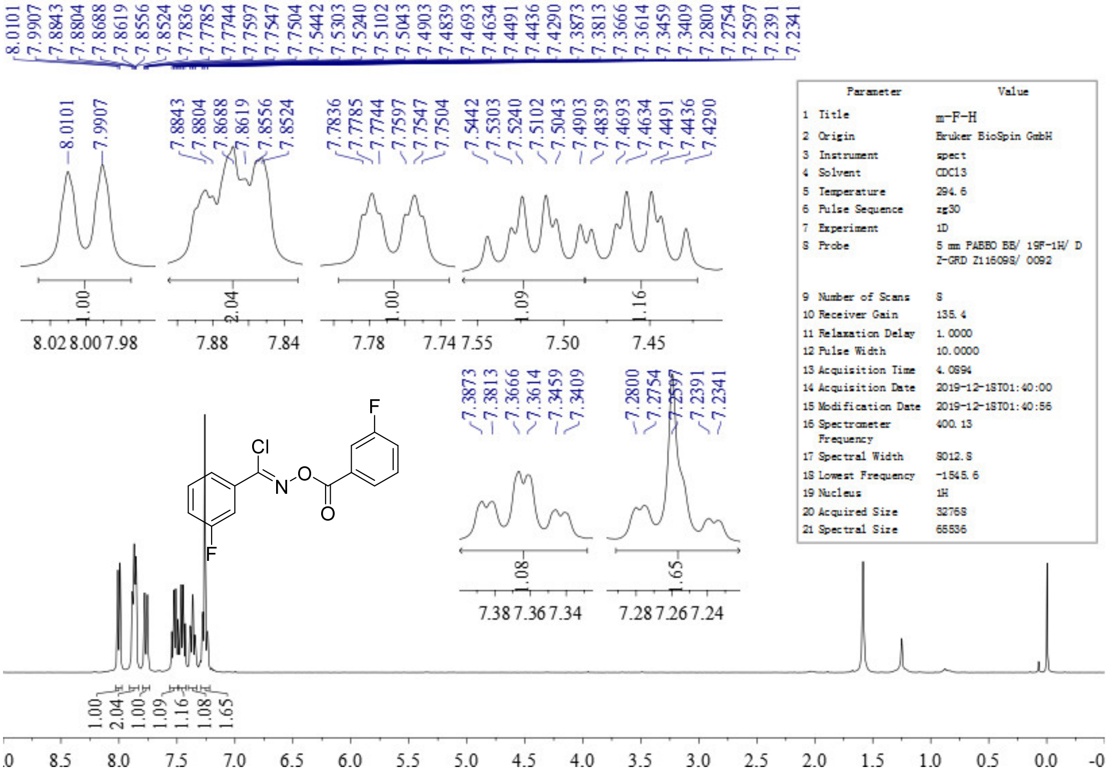

Figure S19. ^{1}H NMR (400 MHz, $CDCl_3$) spectrum of compound 2i.

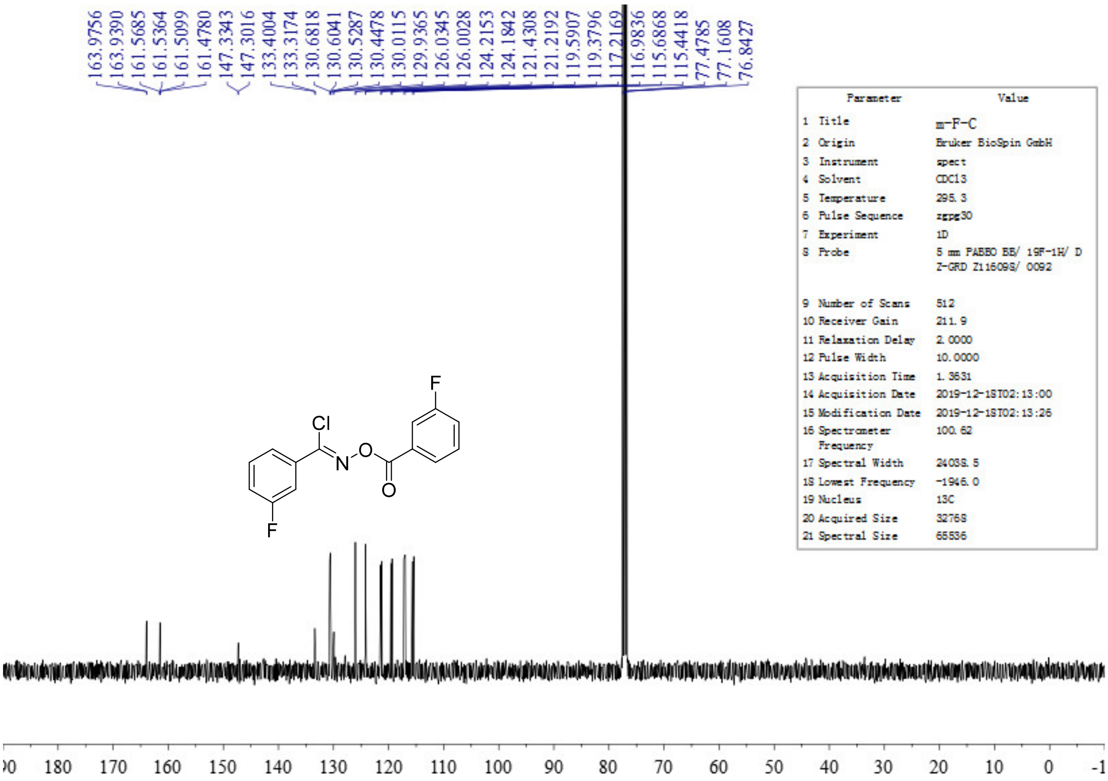

Figure S20. ^{13}C NMR (101 MHz, $CDCl_3$) spectrum of compound 2i.

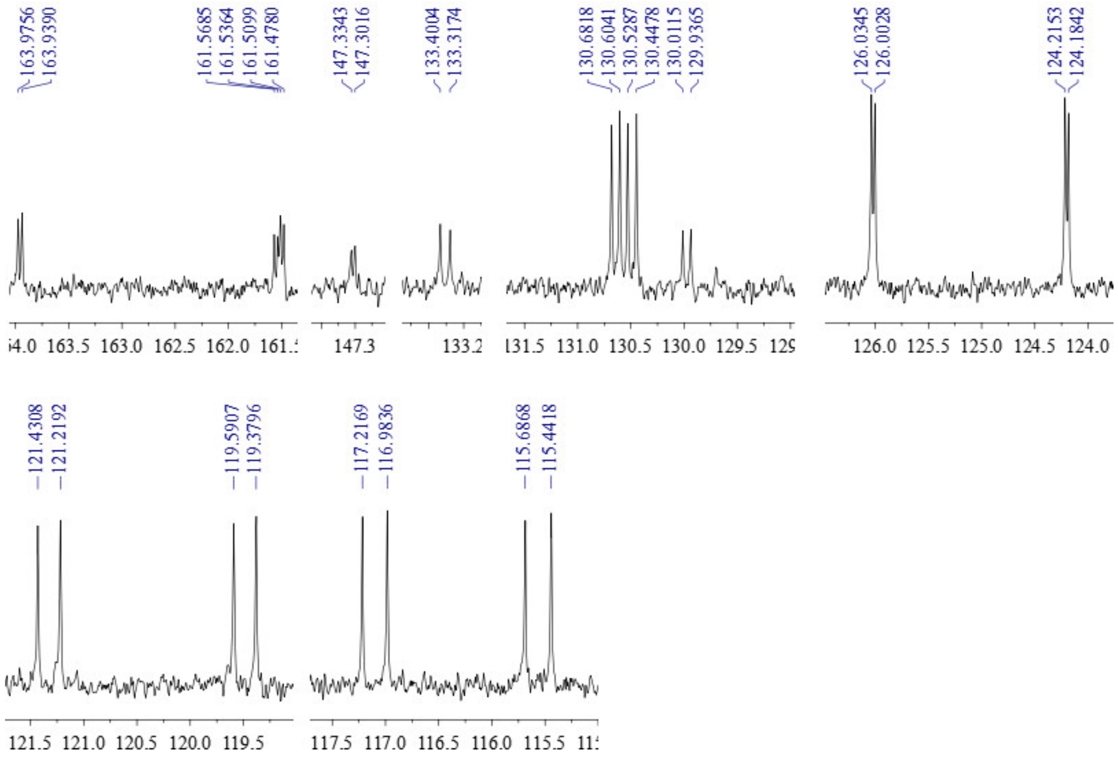

Figure S21. Expanded ^{13}C NMR (101 MHz, $CDCl_3$) spectrum of compound 2i.

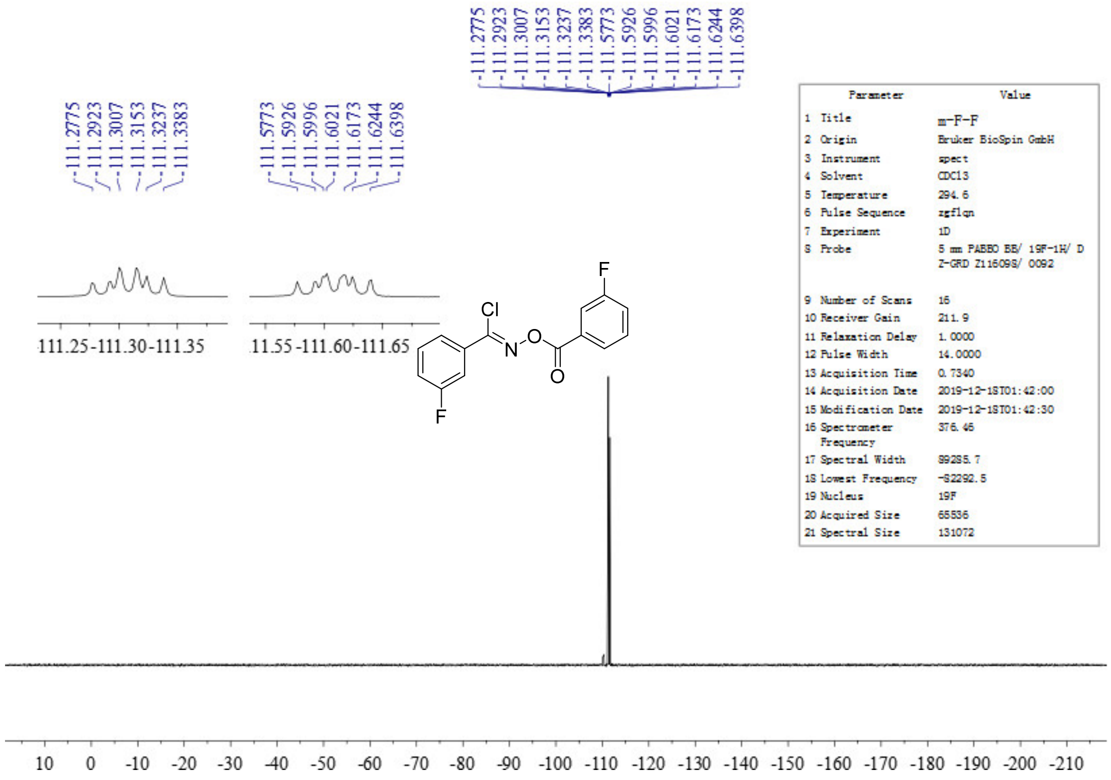

Figure S22. ^{19}F NMR (376 MHz, $CDCl_3$) spectrum of compound 2i.

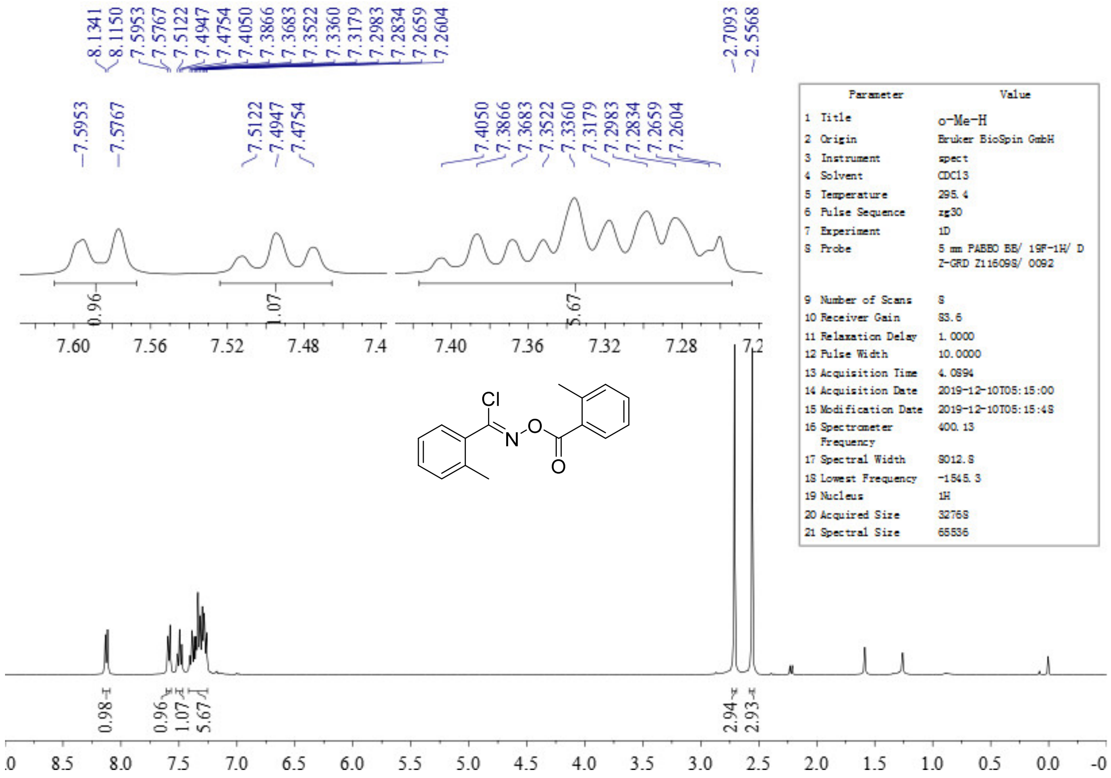

Figure S23. ^{1}H NMR (400 MHz, $CDCl_3$) spectrum of compound 2j.

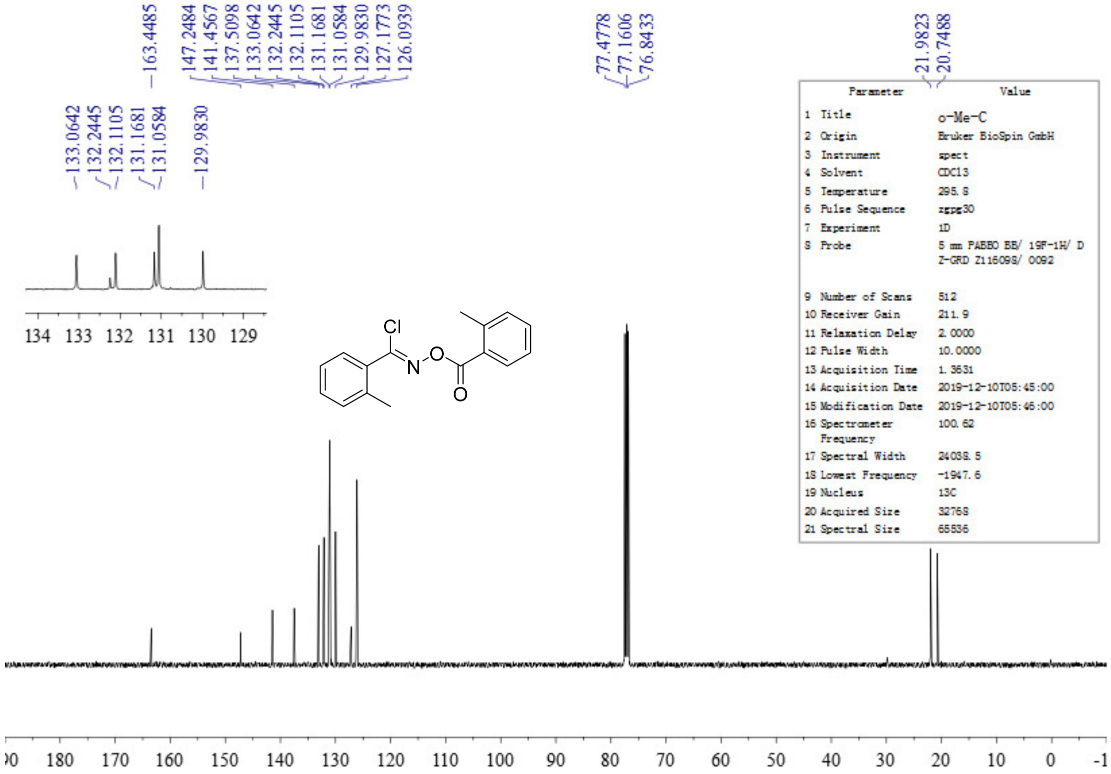

Figure S24. ^{13}C NMR (101 MHz, $CDCl_3$) spectrum of compound 2j.

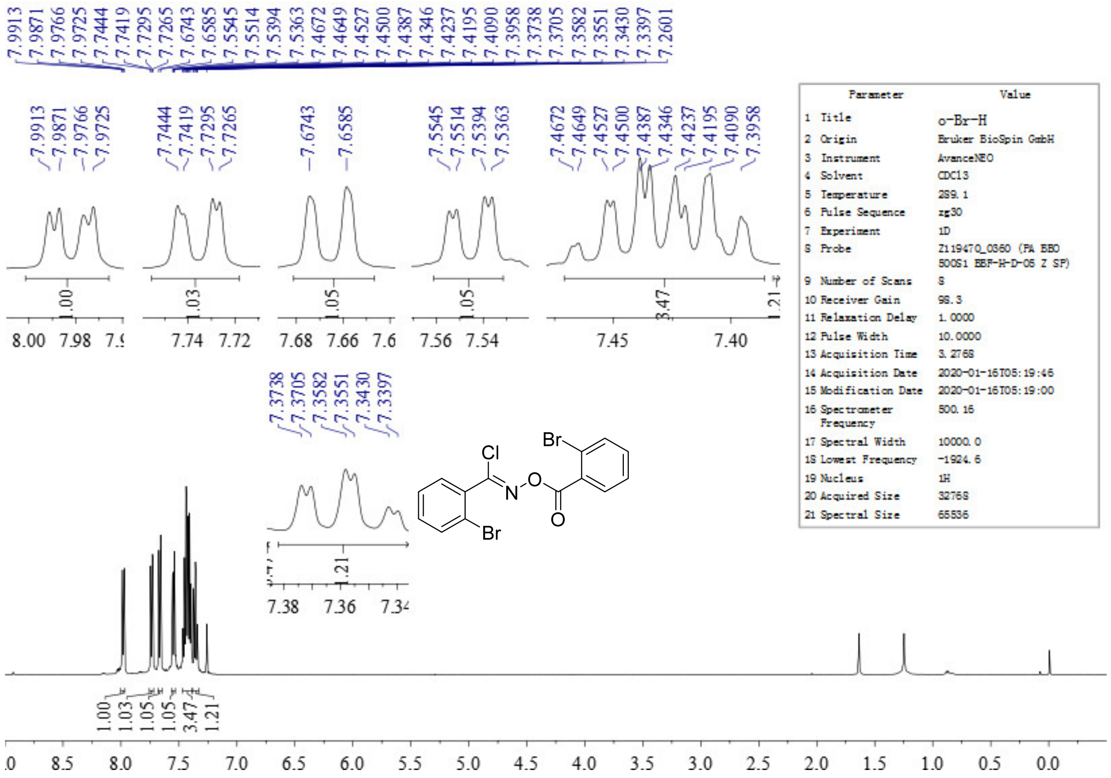

Figure S25. ^{1}H NMR (500 MHz, $CDCl_3$) spectrum of compound 2k.

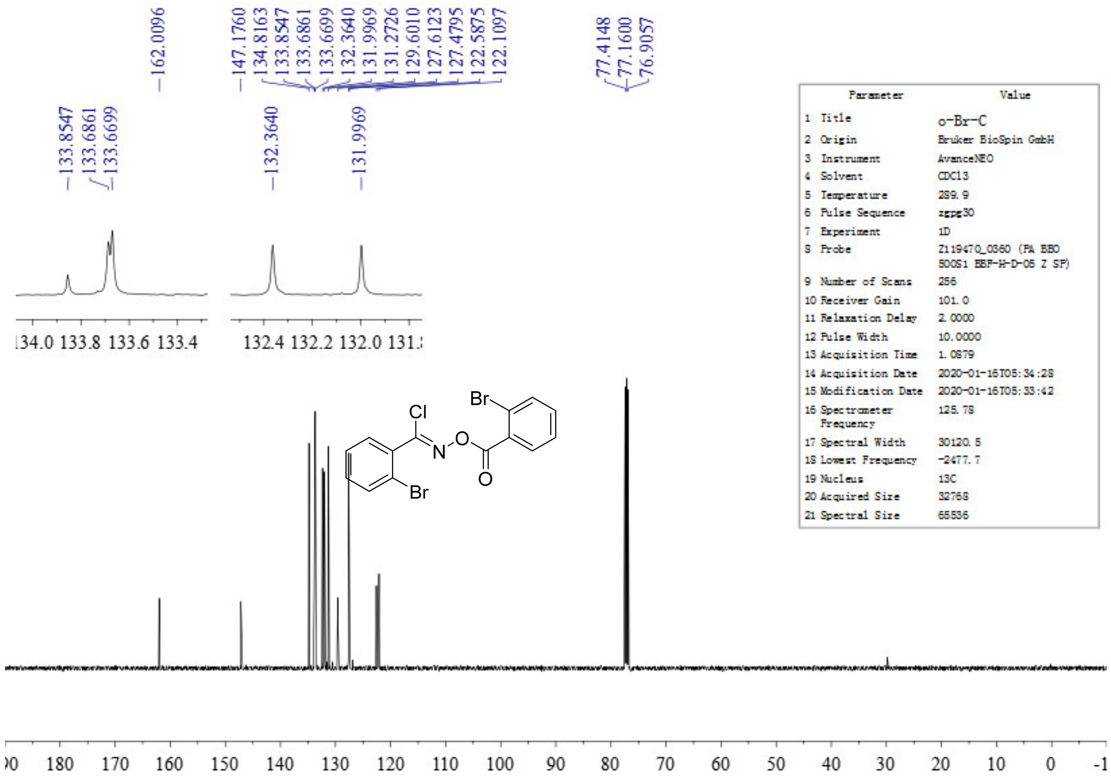

Figure S26. ^{13}C NMR (126 MHz, $CDCl_3$) spectrum of compound 2k.

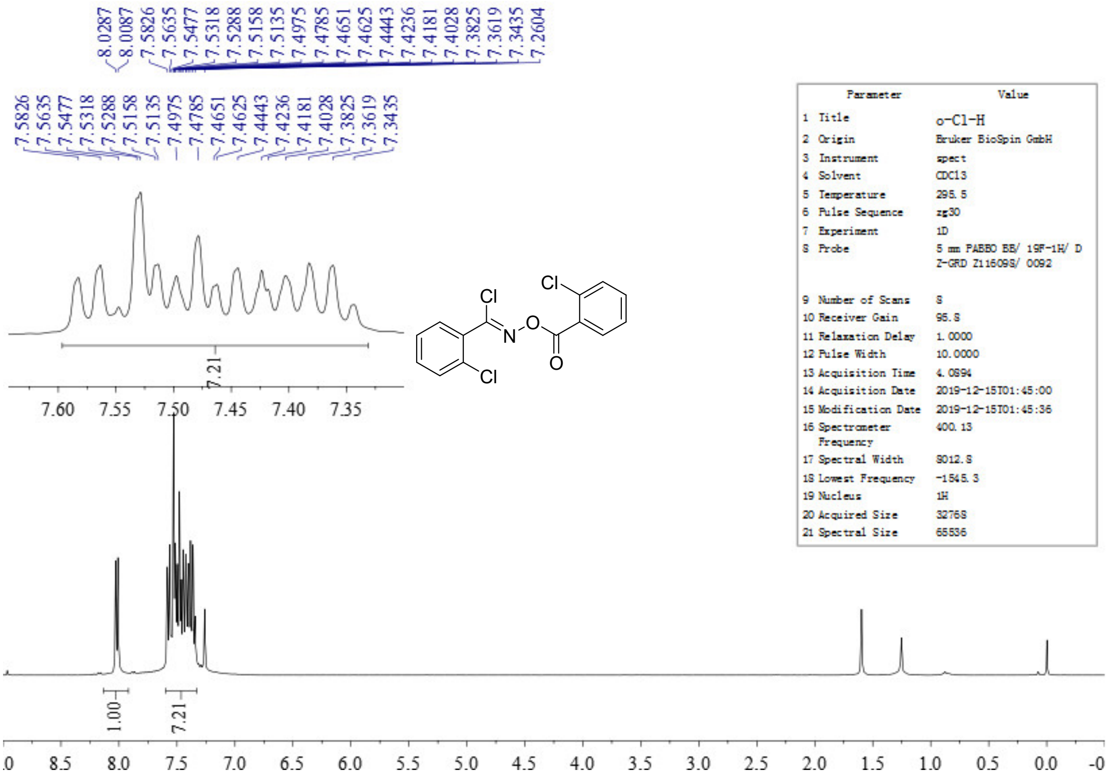

Figure S27. ^{1}H NMR (400 MHz, $CDCl_3$) spectrum of compound 2l.

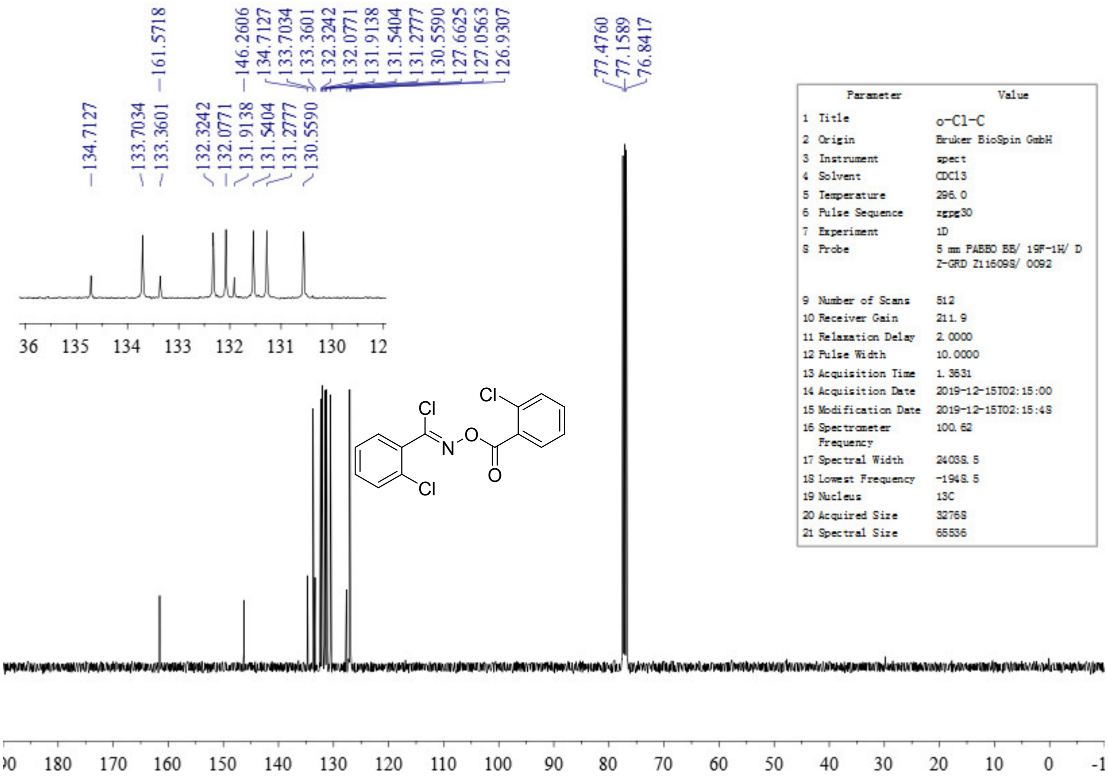

Figure S28. ^{13}C NMR (101 MHz, $CDCl_3$) spectrum of compound 2l.

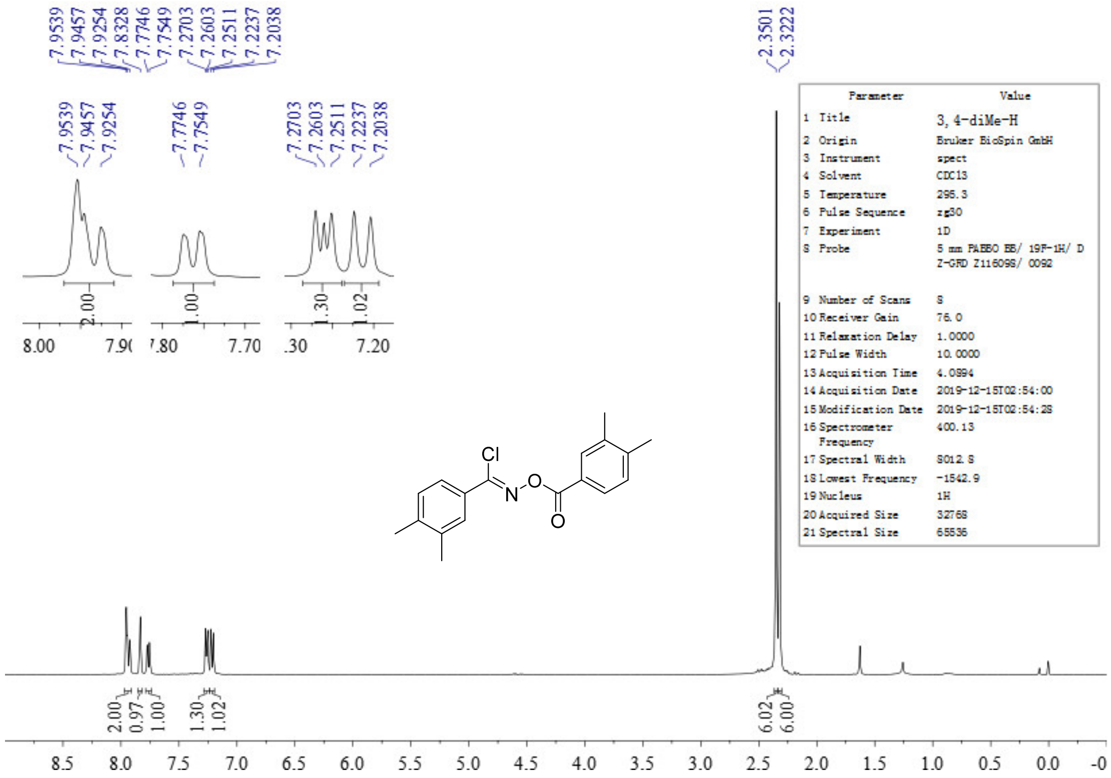

Figure S29. ^{1}H NMR (400 MHz, $CDCl_3$) spectrum of compound 2m.

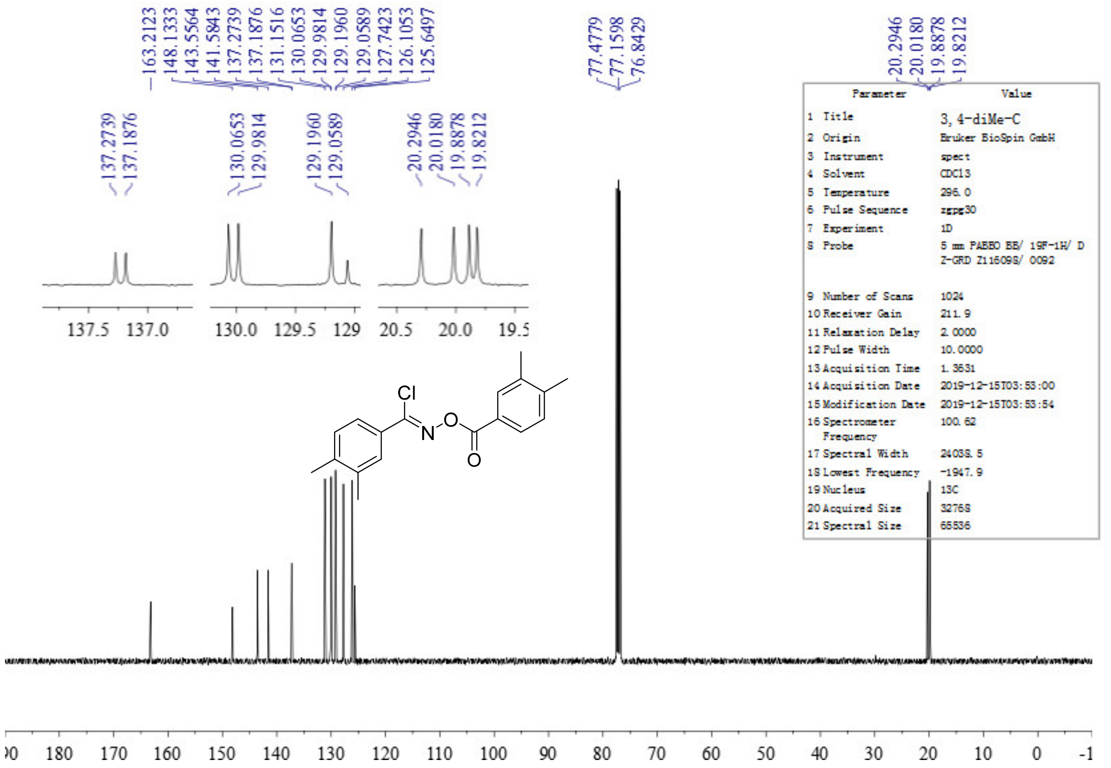

Figure S30. ^{13}C NMR (101 MHz, $CDCl_3$) spectrum of compound 2m.

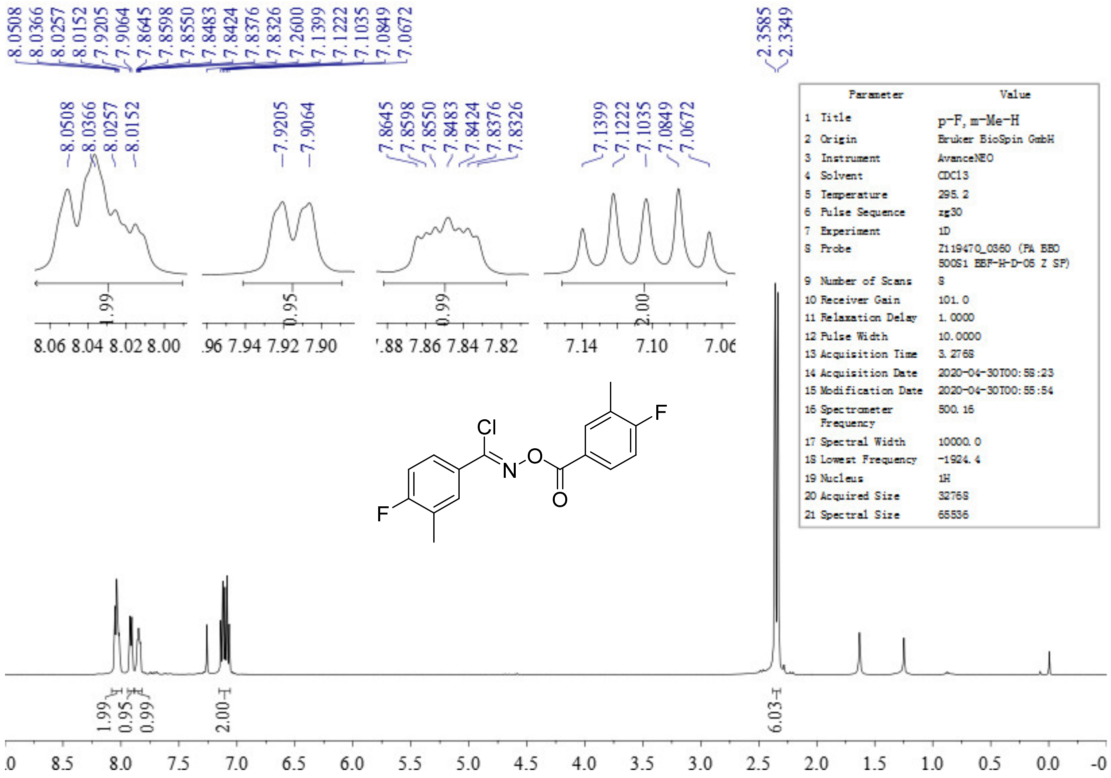

Figure S31. ^{1}H NMR (500 MHz, $CDCl_3$) spectrum of compound 2n.

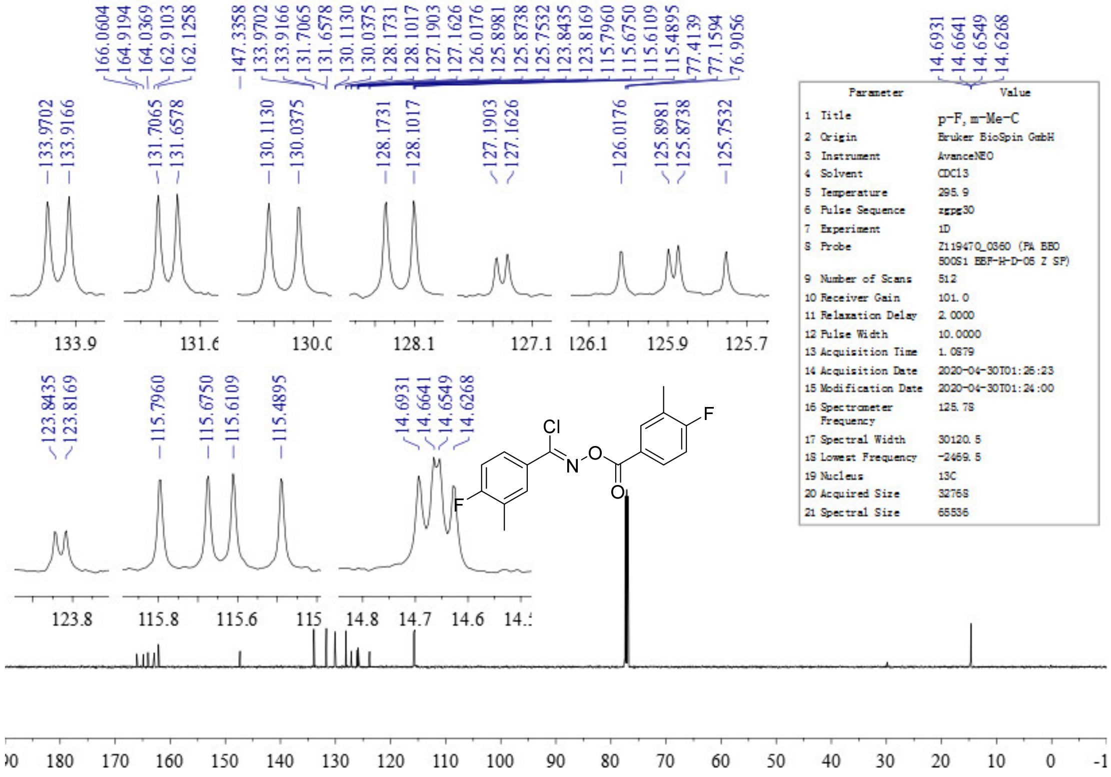

Figure S32. ^{13}C NMR (126 MHz, $CDCl_3$) spectrum of compound 2n.

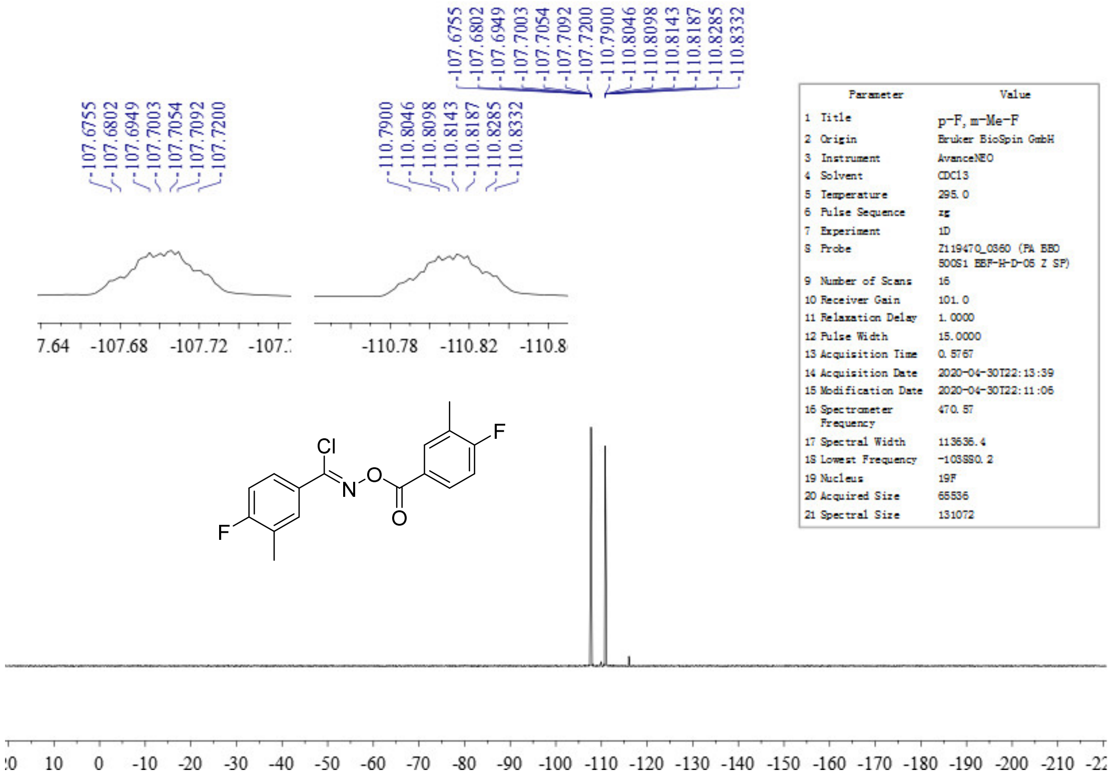

Figure S33. ^{19}F NMR (471 MHz, $CDCl_3$) spectrum of compound 2n.

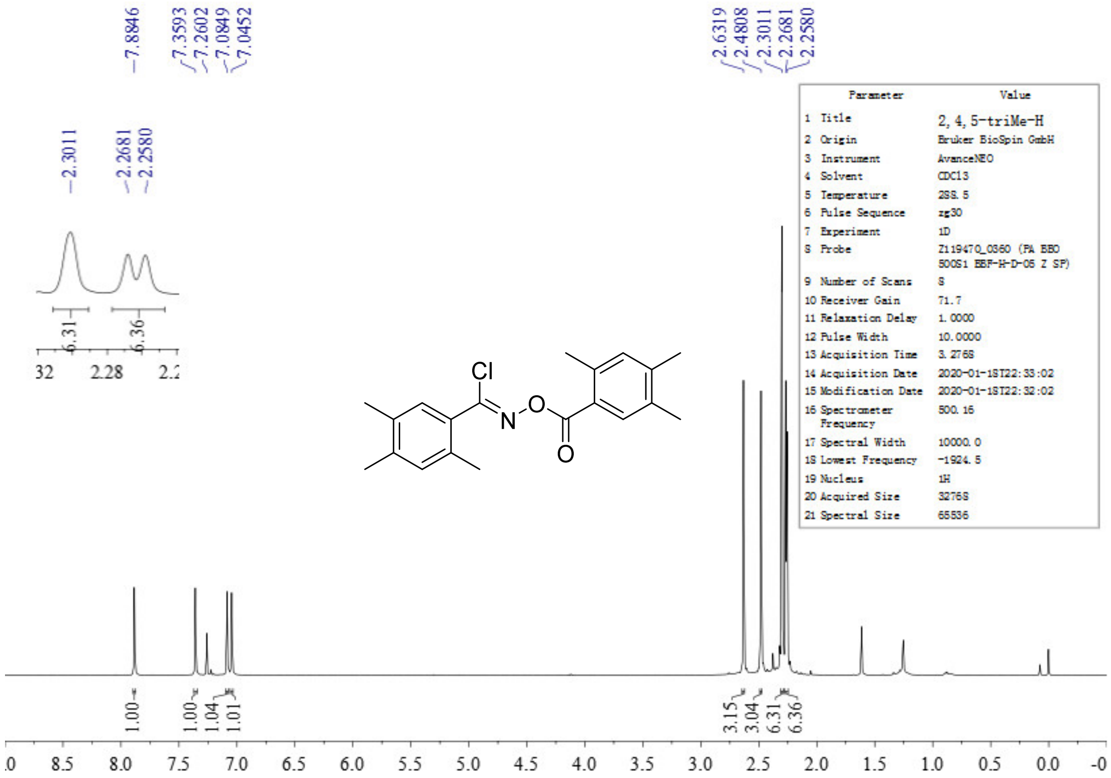

Figure S34. ^{1}H NMR (500 MHz, $CDCl_3$) spectrum of compound 2o.

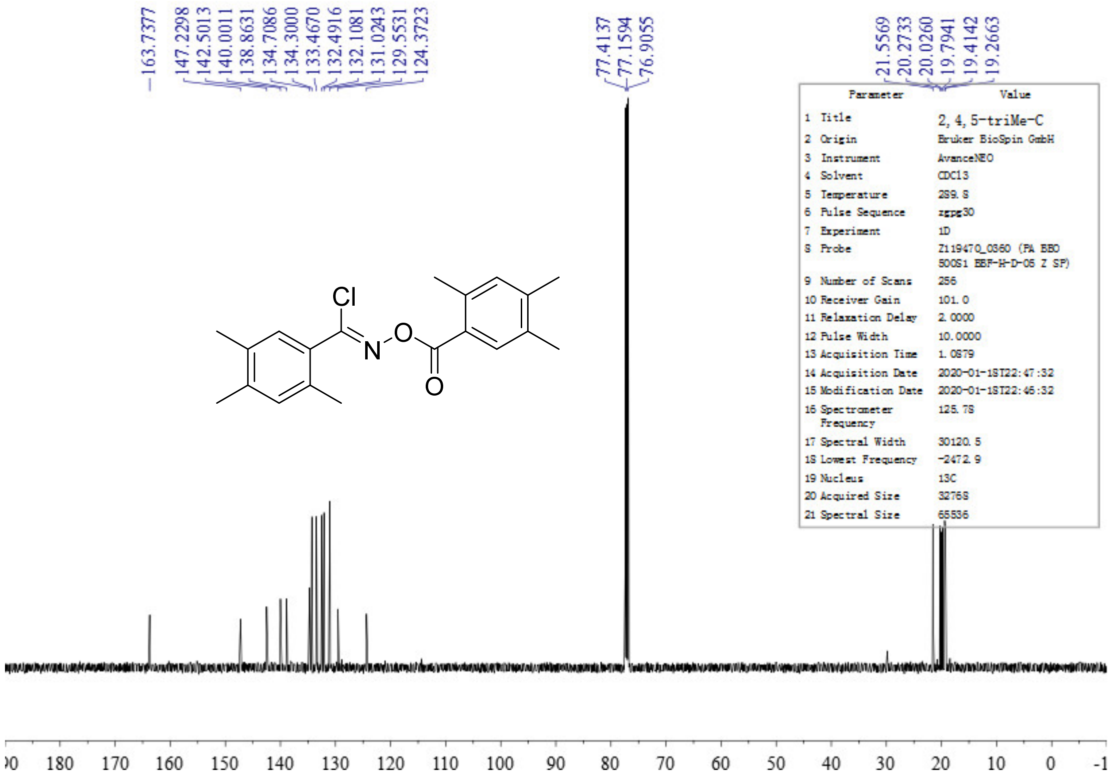

Figure S35. ^{13}C NMR (126 MHz, $CDCl_3$) spectrum of compound 2o.

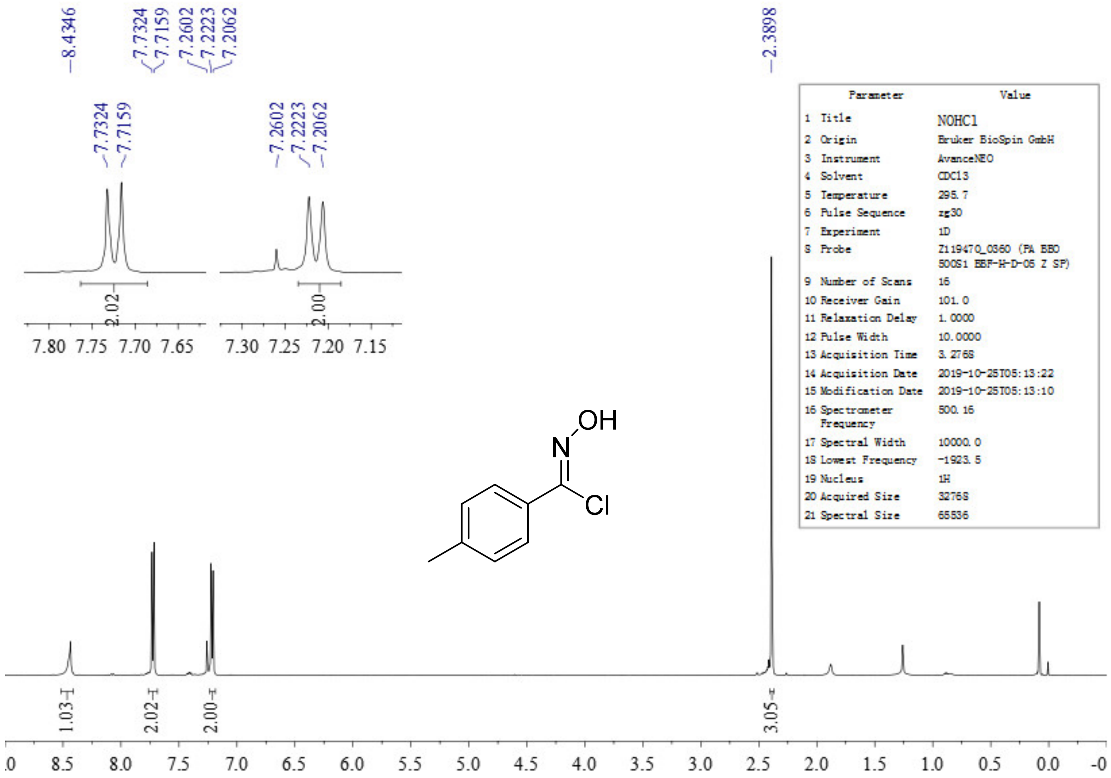

Figure S36. ^{1}H NMR (500 MHz, $CDCl_3$) spectrum of compound 3a.

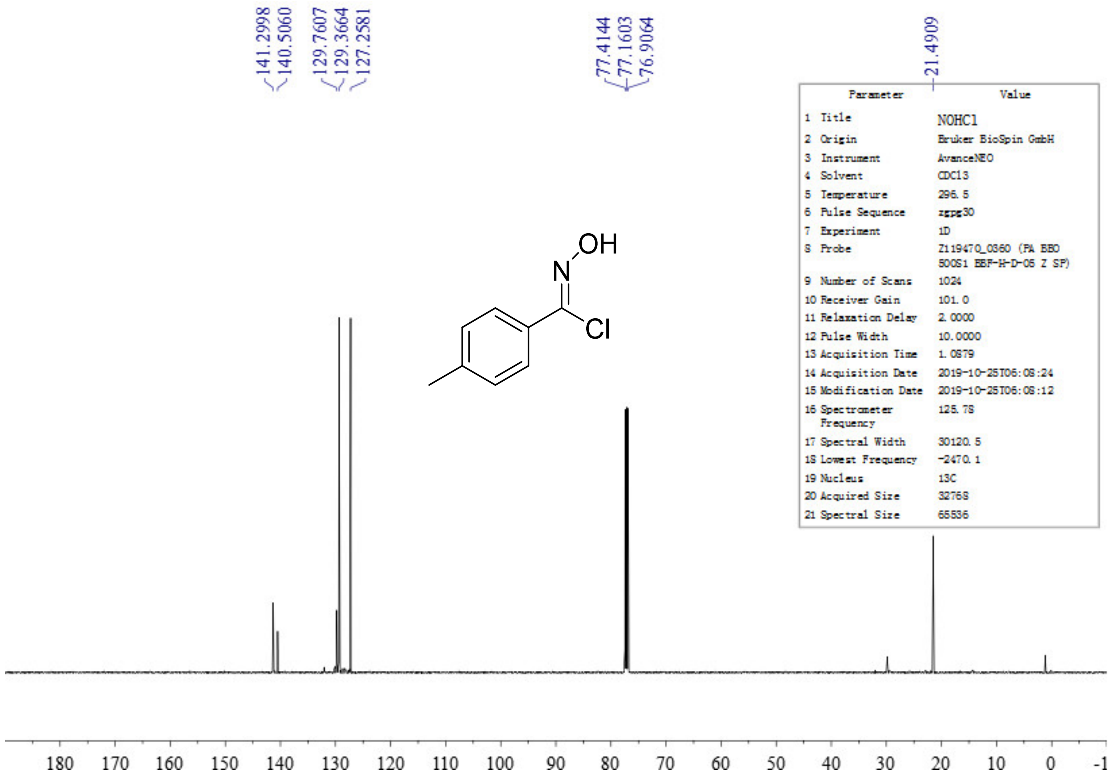

Figure S37. ^{13}C NMR (126 MHz, $CDCl_3$) spectrum of compound 3a.

2. Single-Crystal X-ray Crystallography of 2a

Single crystals of **2a** were obtained by slow evaporation from a mixture of dichloromethane/*n*-hexane at 4 °C. Single-crystal X-ray diffraction data were collected on a diffractometer (Gemini S Ultra, Agilent Technologies) equipped with a CCD area detector using graphite-monochromated Cu Kα radiation (λ = 1.54184 Å) in the scan range 9.254° < 2θ < 146.750°. The structure was solved with direct methods using SHELXS-97 and refined with full-matrix least-squares refinement using the SHELXL-97 program within OLEX2. Crystallographic data have been deposited in the Cambridge Crystallographic Data Centre as deposition number CCDC 2003914.

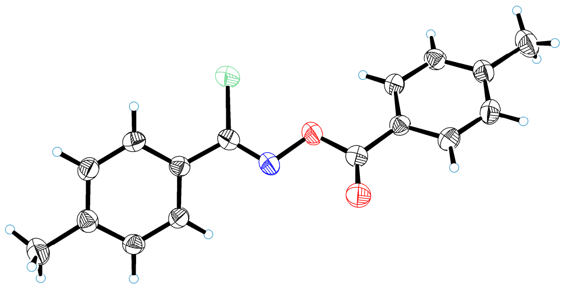

Figure S38. ORTEP Diagrams of **2a** with 30% Thermal Ellipsoids.

Table S1. Crystal Data and Structure Refinement for 2a.

**Identification code**	**2003914**
Empirical formula	$C_{16}H_{14}ClNO_2$
Formula weight	287.73
Temperature/K	293(2)
Crystal system	monoclinic
Space group	$P2_1/c$
a/Å	7.3283(3)
b/Å	17.5340(8)
c/Å	11.3990(5)
α/°	90
β/°	90.530(4)
γ/°	90
Volume/Å^3	1464.65(11)
Z	4
ρ_{calc}g/cm^3	1.305
μ/mm^{-1}	2.313
F(000)	600.0
Crystal size/mm^3	0.250 × 0.220 × 0.150
Radiation	CuKα (λ = 1.54184)
2Θ range for data collection/°	9.254 to 146.750
Index ranges	$-7 \le h \le 8$, $-21 \le k \le 21$, $-14 \le l \le 11$
Reflections collected	5918
Independent reflections	2866 [R_{int} = 0.0196, R_{sigma} = 0.0209]
Data/restraints/parameters	2866/0/183
Goodness-of-fit on F^2	1.059
Final R indexes [I >= 2σ (I)]	R_1 = 0.0496, wR_2 = 0.1441
Final R indexes [all data]	R_1 = 0.0568, wR_2 = 0.1551
Largest diff. peak/hole/e Å^{-3}	0.33/−0.36
